# Effects of rot-promoting bacteria on decomposition characteristics of corn straw and spring soybean yield in Saline-alkali Land

**DOI:** 10.3389/fpls.2025.1572868

**Published:** 2025-05-13

**Authors:** Yuqing Wang, Mingcong Zhang, Lei Lu, Chen Wang, Jianing Wang, Yang Hu, Siyan Li, Wei Xie, Xiwen Hu, Haiqing Guo

**Affiliations:** ^1^ College of Agronomy, Heilongjiang Bayi Agricultural University, Daqing, China; ^2^ Key Laboratory of Low-carbon Green Agriculture in Northeastern China, Ministry of Agriculture and Rural Affairs P. R. China, Daqing, China; ^3^ Department of Foreign Language, Kunlun Tourism College, Harbin, China; ^4^ Comprehensive Service Center of Nadanbo Town, Liaoyuan, Jilin, China; ^5^ Comprehensive Service Center of Agricultural Machinery, Daqing, Heilongjiang, China

**Keywords:** straw, microbial inoculants, decomposition effect, nutrient release rate, extracellular enzyme activity, soybean yield

## Abstract

Understanding the relationship between microbial inoculants and straw decomposition is crucial for achieving a high soybean yield in northern China’s cold region. This study investigated the effects of different microbial inoculants on nutrient release characteristics and extracellular enzyme activities. A pot experiment was conducted over two growing seasons (2023 and 2024) using the soybean (*Glycine* max L. Merrill) cultivar Nongqing 28, the saline-alkali soil as the test soil, and corn straw as the test straw. The microbial inoculants tested were *Bacillus* sp. *ND1* and *Bacillus* sp. *ND2*. The following treatments were employed: straw with no microbial agent application (CK), straw with *Bacillus* sp. *ND1* application (T1), straw with *Bacillus* sp. *ND2* application (T2), and straw with a 1:1 application of Bacillus sp. *ND1* and *Bacillus* sp. *ND2* compound bacteria (T3).The two-year results showed that the T1, T2, and T3 treatments significantly increased the rate of straw decomposition, reduced the lignocellulose content, and progressively released nitrogen, phosphorus, and potassium from the straw compared to the CK. During both years, the T3 treatment exhibited the highest straw decomposition rate and enzyme activity at R2(Full Bloom period), R4(Full Pod period), R6(Full Seed period) and R8(Full Maturity period) periods, which ultimately increased soybean yield by 24.00%-28.00% (*P*<0.05). These findings indicate that microbial inoculants have significant potential for application in straw management and provide an important basis for optimizing straw return and crop yield. In summary, T3 treatment can accelerate straw decomposition and nutrient release rates, increase soybean yield, and provide a theoretical basis for optimizing the straw decomposition effect and rational utilization of organic resources by promoting the activity of extracellular enzymes and the degradation of straw cellulose, hemicellulose, and lignin.

## 1 Introduction

According to related statistics, the world’s annual straw production is about 7 billion tons, while China’s annual straw production accounts for one-seventh of the world’s total production, which means China is a large agricultural country with abundant straw resources. (Sun et al., 2024; Niu and Ju, 2017)., However, more than 200 million tons of straw are burned annually due to the lack of treatment and utilization technologies. In other words, its loss of nitrogen, phosphorus, and potassium is equivalent to about 60% of the total output of fertilizers in this country (Hua et al., 2010), resulting in resource wastage (Zhai et al., 2012) and environmental pollution (Cheng et al., 2025; Li et al., 2018). Therefore, it is necessary to promote straw decomposition with appropriate technical methods in current agriculture.

In general, the slow decomposition rate of straw is closely related to its complex chemical composition and stable physical structure (Liu et al., 2015). The whole process is as follows. Specifically, the waxy layer on the surface of straw is hydrophobic, which limits the penetration of water and microorganisms. Additionally, straw contains a significant amount of lignin, whose complex aromatic structure forms a natural protective barrier that encases cellulose and hemicellulose, thereby hindering microbial and enzymatic access for decomposition. Cellulose is tightly organized within the crystalline zone, making it resistant to degradation by cellulase, while the heterogeneous nature of hemicellulose further complicates degradation. In Northeast China, prolonged low temperature conditions inhibit the activity of decomposing microorganisms. Furthermore, corn straw, which constitutes the majority of returned straw in this region, possesses vascular bundles and a thick-walled cell structure that are extremely dense and exhibit high mechanical strength (Ninkuu et al., 2025). Consequently, this results in low efficiency of straw decomposition, which has become the primary bottleneck limiting the utilization of straw resources in Northeast China.

The application of microbial inoculants can improve the structure and function of soil microbial communities. Because the relative abundance of cellulose-degrading bacteria in the soil increased significantly after inoculation, the strains promoted the decomposition of straw by secreting cellulase and lignin-degrading enzymes (Zhao et al., 2024), thereby advancing the release of nutrients from straw. In short, inoculation of functional microorganisms further improved cellulase and xylanase activities, significantly releasing efficiency of organic carbon and nutrients in straw (Wu et al., 2025).

Degradation of lignocellulose requires the coordination of several hydrolytic enzymes, including endoglucanase, exoglycanase, β-glucosidase, and xylanase (Priyanka et al., 2024). Endo-glucanase disrupts the crystalline structure of cellulose by randomly cleaving the β-1,4-glycosidic bonds within cellulose to generate new chain ends. Exo-glucanase acts mainly on the ends of cellulose chains, releasing cellobiose one by one. The activity of this enzyme closely relates to the crystalline structure of cellulose. Exo-glucanase acts mainly on the ends of cellulose chains, releasing cellobiose one by one. The activity of this enzyme is usually closely related to the crystalline structure of cellulose (Chen et al., 2025) β-glucosidase further hydrolyzes cellobiose to glucose, which is a key enzyme in cellulose degradation. Its action is effective in reducing the accumulation of cellobiose, thereby increasing the overall efficiency of cellulose degradation. Xylanase is an enzyme that mainly acts on the xylan in hemicellulose and decomposes xylan into soluble sugars by hydrolyzing the β-1,4-xyloside bond (Chen et al., 2025). Xylanases play an important role in lignocellulose degradation. They can synergize with the cellulase system to improve the overall degradation efficiency (Li T. et al., 2024).

Cellulose degrading microorganisms dominate the current research on straw degrading bacterial agents (Zhao et al., 2024). Researchers at home and abroad have screened several strains with cellulose degrading ability, including bacteria, fungi, and actinomycetes from the natural environment (Zhao et al., 2017). Bacteria that have been isolated include Pseudomonas genus, Rhizobium genus, Cellulomonas genus, Bacillus genus, etc. (Awasthi et al., 2017; Wang et al., 2017). Straw degradation results from the synergistic action of multiple enzyme systems because a single strain is difficult to produce a more complete enzyme system. So, the ability of straw degradation is limited. However, various strains can produce various enzyme systems after the composite, improving the degradation effect (Lynd et al., 2002). At this stage, most studies focus on straw-rotting bacteria in acidic or neutral soils, while less research has been done on saline and alkaline soils. The two Bacillus species selected for the experimental treatment based in the condition that there is no antagonistic reaction between the strains in the early stage in the laboratory. With the straw decomposition test finishing, the results showed that the decomposition effect of these two fungal agents on the straw is ideal. Thus, we want to take the Bacillus to the pot experiment. If the pot test effect is ideal, the author would apply it in the field test in the next step. Given this, this experiment used the previously screened Bacillus as the test material to explore the relationship between different decomposition agents on straw decomposition, nutrient release characteristics and extracellular enzyme activity changes in Northeast China, which would provide a basis for the utilization of low-temperature straw decomposition bacteria in Northeast China.

## 2 Materials and methods

### 2.1 Experimental site conditions

The experiment was carried out in the potting field on the campus of Heilongjiang Bayi Agricultural University in 2023-2024. The test site was in Daqing City, Heilongjiang Province (between 46°38′N latitude and 125°14′E longitude), which is located in the western part of the Songnen Plain in Northeast China. The climate here is cold in winter, windy in spring and autumn, with less precipitation, a short frost-free period throughout the year, rain and heat in the same season, an average annual temperature≥10 °C, an annual effective accumulated temperature of 2000°C-2300°C, and a frost-free period of 115-120d, which belongs to the cold temperate continental climate.

### 2.2 Experimental materials

The Soybean (Glycine max L. Merrill) cultivar for test: Nongqing 28, sub-limited podding habit, about 123 days of fertility. No branches, white flowers, round leaves, gray velvet, pods curved, sickle-shaped, brown at maturity, seeds round, seed coat yellow, seed umbilicus yellow, shiny. Fertilizers for the test are urea (N≥46%) and ammonium sulfate (N≥21%), diammonium phosphate (N≥18%, P2O5≥46%), and potassium magnesium sulfate (K2O≥24%, Mg≥6%). All the above materials belong to marketed production materials. Bacteria for test: microbial inoculants 1 (Bacillus sp. ND1, 4×10^7 CFU/ml), microbial inoculants 2 (Bacillus sp. ND2, 4×10^9 CFU/ml). Soil for test: saline soil. (taken from 0-20 cm topsoil around Daqing City with physicochemical properties as shown in **Table 1**) Straw for test: corn straw. (Initial nutrients are shown in **Table 2**.)

**Table 1:** Physicochemical properties of the test soil.

Year	pH	Organic matter	AP P_2_O_5_ (mg/kg)	AK K_2_O (mg/kg)	AN N (mg/kg)	Conductivity (μS/cm)
2023	8.73	25.47	28.40	136.67	92.06	420.17
2024	8.95	31.29	29.50	118.00	123.17	464.00

**Table 2:** Initial nutrient content of test straw.

Year	TN	TP	TK	TC	C/N	Cellulose	Hemicellulose	Lignin
	(mg/g)	(mg/g)	(mg/g)	(mg/g)	(mg/g)	(mg/g)	(mg/g)	(mg/g)
2023	8.03	2.25	15.45	614.8	77.0	281.5	352.0	158.7
2024	5.00	6.87	24.44	356.7	72.0	327.5	369.0	229.5

### 2.3 Design of experiments

Four treatments were established: The CK, which involved straw with no microbial agent application; the T1 treatment, which included straw with applying Bacillus sp. ND1; T2 treatment utilized straw with the application of Bacillus sp. ND2 and T3 treatment consisted of straw with a 1:1 mixture of Bacillus sp. ND1 and Bacillus sp. ND2. Each treatment replicates 3 times. The experiment was conducted using the nylon mesh bag burial method, which involved several steps: first, 65g of dried straw (3 cm to 5 cm in length) was placed in a nylon mesh bag (25 cm × 15 cm); the straw was then soaked in either Bacteria 1 (4 × 10^7 CFU/ml), Bacteria 2 (4 × 10^9 CFU/ml), or the mixture of Bacteria 1 and Bacteria 2. After the straw was thoroughly wetted, the bag was buried in a potting bucket (45 cm in height and 15 cm in diameter) at a depth of 25 cm prior to mulching. Urea water was then sprayed according to the carbon-to-nitrogen (C/N) ratio of the straw, adjusted to 30:1 while maintaining the soil moisture content at approximately 60% to 70% of the field's water-holding capacity throughout the experimental period. Fertilizer application was calculated based on conventional soybean production practices in the region, with total applications of nitrogen (N) at 60 kg/hm², phosphorus pentoxide (P2O5) at 70 kg/hm², and potassium oxide (K2O) at 30 kg/hm². This included the commercial amounts of urea at 32.61 kg/hm², ammonium sulfate at 85.71 kg/hm², diammonium phosphate at 150 kg/hm², and potassium magnesium sulfate at 60 kg/hm², with nitrogen, phosphorus, and potassium fertilizers applied as a single base application. Additionally, the nitrogen, phosphorus, and potassium amounts applied across each treatment were kept consistent. Finally, all other field management practices adhered to local production standards. In summary, sowing occurred in May, and harvesting occurred in September.

### 2.4 Measurement items and methods

Straw decomposition samples and soil samples were collected during different soybean periods, which sampled from R2(Full Bloom period), R4(Full Pod period), R6(Full Seed period) and R8(Full Maturity period). Six pots were taken each time. Three pots of straw decomposition samples were repeatedly rinsed with deionized water to remove the soil on the surface and dried at 60°C until constant weight then determined the decomposition rate of straw, and finely ground through a 0.25mm sieve to determine the nutrients, cellulose, hemicellulose, and lignin of the straw. In the meantime, the experimenter took three pots of other samples, directly opened the nylon bag, carefully removed the straw and then cut it into pieces, and stored it at 4°C for the measurement of the extracellular enzyme activities related to straw decomposition.

#### 2.4.1 Straw decomposition rate


$$\text{Straw decomposition rate}\left(\%\right)=\frac{\text{W}1-\text{W}2}{\text{W}1}\times 100\%$$

W_1_ is the original straw weight (g); W_2_ is the weight of straw after decomposition in different sampling periods (g).

#### 2.4.2 Straw nutrient release rate

The total nitrogen content of straw was determined by Kjeldahl method; the total phosphorus content of straw was determined by vanadium-molybdenum yellow colorimetric method; and the total potassium content of straw was determined by flame spectrophotometer method (Bao, 2000).


$$\text{Nutrient release rate}=\left({\text{M}}_{0}{\text{C}}_{0}-{\text{M}}_{\text{t}}{\text{C}}_{\text{t}}\right)/{\text{M}}_{0}{\text{C}}_{0}\times 100\%$$

M0 is the initial dry weight of straw (g); C0 is the initial content of straw nutrients (mg/g); Mt is the dry weight of straw at time t of decomposition (g); Ct is the nutrient content of straw at time t of decomposition(mg/g); t is the sampling period.

#### 2.4.3 Lignin, cellulose, and hemicellulose of straw

Cellulose, hemicellulose and lignin in straw were determined by the modified Van Soest washed fibres method (Yang, 1993).

#### 2.4.4 Extracellular enzyme activity of straw

exo-β-1, 4-glucanase, endo -β-1,4-glucanase, xylanase and β-mannanase were determined by kits provided by Suzhou Geruisi Biotechnology Limited Company (China).

exo-β-1,4-glucanase, endo-β-1,4-glucanase, xylanase and β-mannanase: Weigh 0.2 g of straw, add 1 ml of pre-cooled 95% ethanol ice bath homogenization, and place at 4°C for 10 minutes; 12,000 rpm, centrifugation at 4°C for 5 minutes; discard the top please, leave the precipitate, add pre-cooled 80% ethanol to the precipitate mixing, and place at 4°C for 10 minutes; Add pre-cooled 80% ethanol to the precipitate and mix well. Add 1 ml of pre-cooled extract to the precipitate, vortex and mix well, leave at 4°C for 10 minutes; centrifuge at 12000 rpm for 10 minutes; leave the supernatant and discard the precipitate. The supernatant was put on ice to be measured. The enzyme counter was warmed up for 30 min, and the wavelength was adjusted to 540 nm. 200 μl of the clarified liquid was taken into a 96-well plate, and the absorbance value A was read at 540 nm, and ΔA was calculated (assay tube - control tube, and one control tube was made for each sample), and the result was calculated in the formula.

exo-β-1, 4-glucanase, endo -β-1,4-glucanase, xylanase and β-mannanase were determined by kits provided by Suzhou Geruisi Biotechnology Limited Company (China).

exo-β-1,4-glucanase, endo-β-1,4-glucanase, xylanase and β-mannanase: Weigh 0.2 g of straw, add 1 ml of pre-cooled 95% ethanol ice bath homogenization, and place at 4°C for 10 minutes; 12,000 rpm, centrifugation at 4°C for 5 minutes; discard the top please, leave the precipitate, add pre-cooled 80% ethanol to the precipitate mixing, and place at 4°C for 10 minutes; Add pre-cooled 80% ethanol to the precipitate and mix well. Add 1 ml of pre-cooled extract to the precipitate, vortex and mix well, leave at 4°C for 10 minutes; centrifuge at 12000 rpm for 10 minutes; leave the supernatant and discard the precipitate. The supernatant was put on ice to be measured. The enzyme counter was warmed up for 30 min, and the wavelength was adjusted to 540 nm. 200 μl of the clarified liquid was taken into a 96-well plate, and the absorbance value A was read at 540 nm, and ΔA was calculated (assay tube - control tube, and one control tube was made for each sample), and the result was calculated in the formula.

#### 2.4.5 Scanning electron microscopy

The surface microstructure of the straw was observed by ultra-high resolution field emission scanning electron microscopy (SEM), using vacuum-dried and sprayed-gold samples, imaging at an accelerating voltage of 10 kV with a magnification of 500 times. Subsequently, the initial straw, CK, T1, T2 and T3 treatments were observed.

#### 2.4.6 Soybean yield and its components

After the soybean matured, five pots per treatment were randomly selected for yield measurement. At the same time, indicators such as the number of pods per plant, the number of grains per plant, the weight of grains per plant, the weight of 100 grains, and the theoretical yield (weight of grains per plant) were determined.

### 2.5 Data analysis

For data analysis, Excel 2019 was used for data collation, and SPSS 25.0 was used for significance analysis. Furthermore, one-way ANOVA and Duncan methods were used for variance analysis and multiple comparison. Data were Plotted through Origin Pro2024b. At last, the partial least squares path model (PLS-PM) and Mantel-test correlation heat map were constructed using the R language.

## 3 Results and analysis

### 3.1 Effect of applying microbial inoculants on the decomposition rate of corn straw

**Figure 1:**
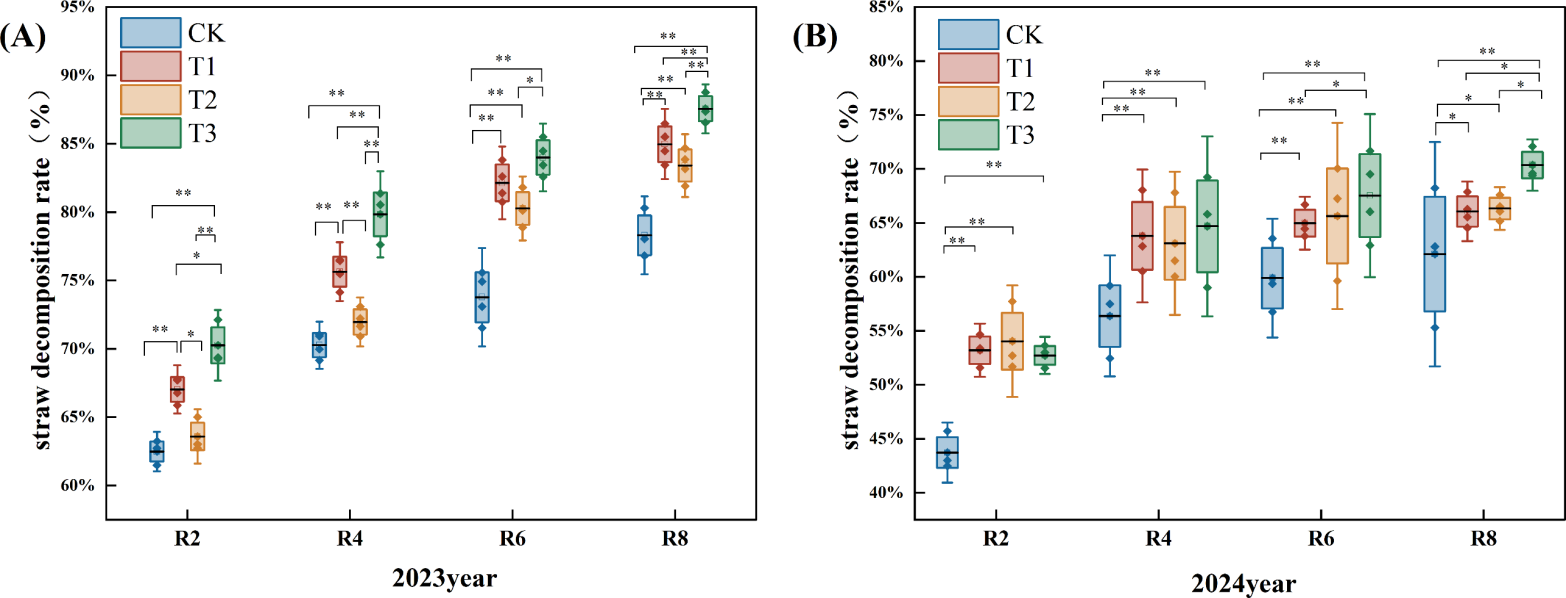
Effects of different exogenous microbial agents on the decomposition rate of straw in two years. **(A)** Pot experiment in 2023. **(B)** Pot experiment in 2024. * and ** in the figure indicate that the correlation reaches the level of 0.05 and 0.01 significant difference, respectively.

**Figure 1** illustrates that the straw decomposition rate escalated as the reproductive period progressed during the two-year experiment. The straw decomposition rate of the T1, T2, and T3 treatments exceeded those of the CK.

In 2023, the decomposition rate of R2, R4, R6, and R8 periods under the T1, T2, and T3 treatments were significantly elevated compared to the CK. The decomposition rate during the R8 period increased by 8.00%, 7.00%, and 12.00% (*P*<0.05). In comparison to the T1 and T2 treatments, T3 treatment at R2, R4, R6, and R8 periods exhibited respective increases in rotting rates of 5.00% and 10.00%; 5.00% and 11.00%; 3.00% and 4.00%; 4.00% and 4.00% (*P*<0.05) (**Figure 1A**).

In 2024, relative to the CK, the T1, T2, and T3 treatments markedly enhanced the decomposition rates at the R2, R4, R6, and R8 periods. The decomposition rate in the R8 period increased by 6.00%, 6.00%, and 13.00%, respectively (*P*<0.05). The decomposition rate of the T3 treatment at the R2 period was inferior to that of the T1 and T2 treatments. The decomposition rate of the T3 treatment at the R8 period was 7.00% and 6.00% higher than that of the T1 and T2 treatments (*P*<0.05). At period R6, the decomposition rate of the T3 treatment was considerably elevated by 5.00% compared to the T1 treatment (*P*<0.05) (**Figure 1B**).

### 3.2 Effect of applying microbial inoculants on lignocellulose of corn straw

**Figure 2:**
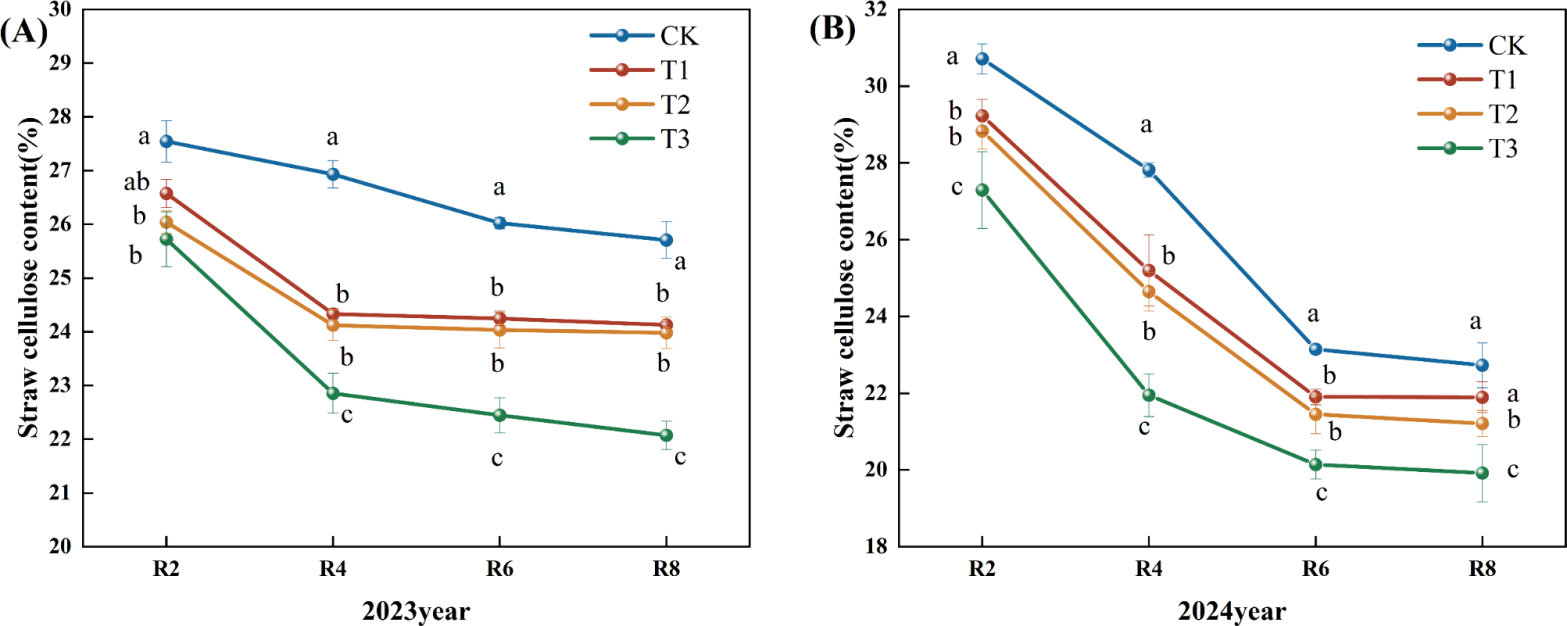
Effects of different microbial agents on cellulose content of corn straw in two years. The 2023 pot experiment. **(B)** The 2024 pot experiment. Different letters represent 5 % significant difference between treatments.

**Figure 2** illustrates that the fast degradation of straw cellulose predominantly occurred during the pre-reproductive phase. In 2023, the cellulose content of each treatment gradually decreased following the peak rate of decline observed in R4 (**Figure 2A**). During the R8 period, the cellulose content of straw in the T1, T2, and T3 treatments diminished by 6.15%, 6.72%, and 14.14%, respectively, compared to the CK. In the R4, R6, and R8 periods, the cellulose content in the T3 treatment was markedly lower than in the other treatments. In 2024, the reduction in cellulose content peaked during the R6 period and subsequently attained its lowest point in the R8 period (**Figure 2B**). Compared to the CK, the cellulose content in the T1, T2, and T3 treatments diminished by 4.00%, 7.00%, and 12.00%, respectively. The cellulose content in the T3 treatment was much lower than in the other treatments in the R2, R4, R6, and R8 periods. Based on the two-year data, the T3 treatment exhibited the lowest straw cellulose concentration and superior decomposition efficacy.

**Figure 3:**
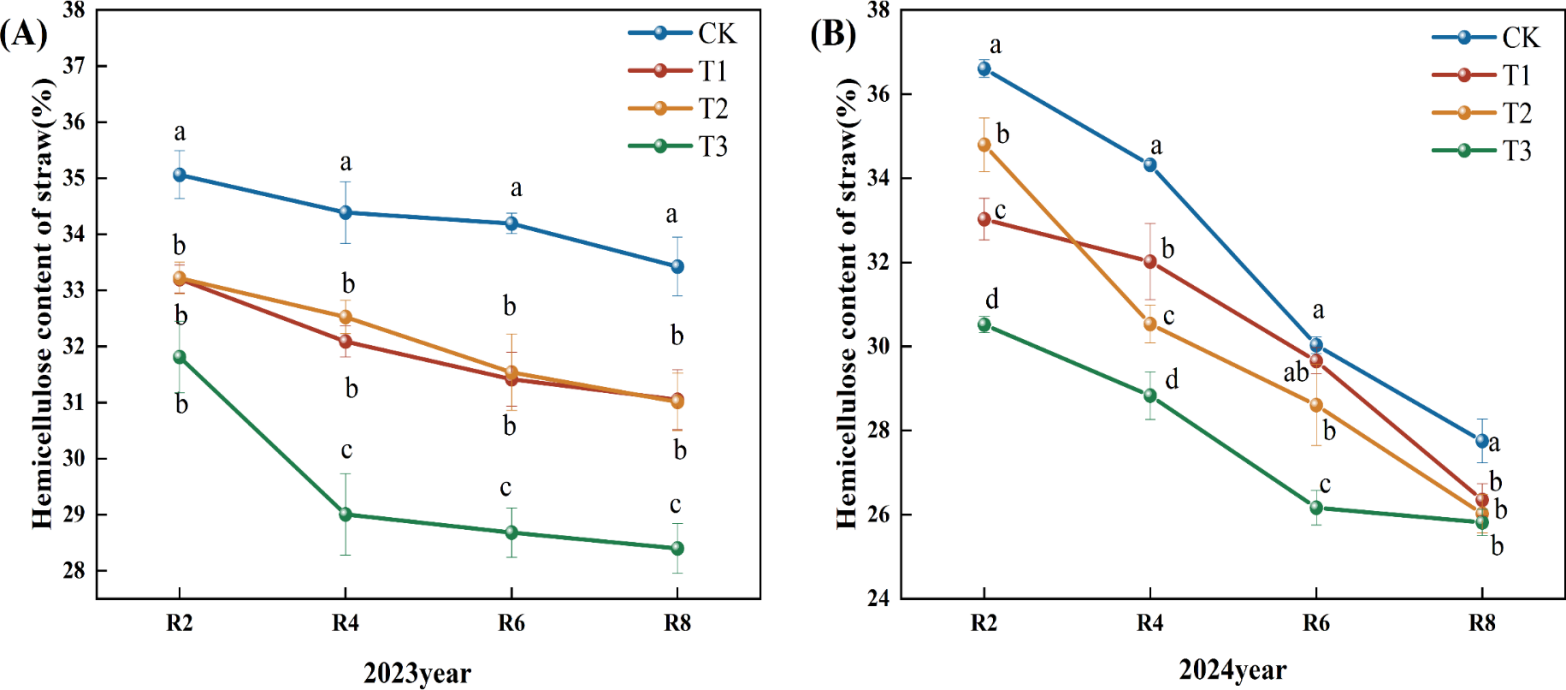
Effects of different microbial agents on hemicellulose content of corn straw in two years. **(A)** The 2023 pot experiment. **(B)** The 2024 pot experiment. Different letters represent 5 % significant difference between treatments.

**Figure 3** reveals that the fast reduction in straw hemicellulose concentration in 2023 predominantly transpired between R2 and R4, followed by a slow decline post-R4. During the R8 period, the straw hemicellulose concentration in the T1, T2, and T3 treatments decreased by 7.10%, 7.22%, and 15.04%, respectively, compared to the CK. During the R4, R6, and R8 periods, the hemicellulose content in the T3 treatment was markedly lower than that of the other treatments (**Figure 3A**). In 2024, there was a gradual decline in straw hemicellulose content; relative to the CK, the hemicellulose content decreased by 5.00% and 13.00% during the R6 period of the T2 and T3 treatments, respectively (P<0.05). At the R8 period, the hemicellulose content in the T1, T2, and T3 treatments decreased by 5.00%, 6.00%, and 7.00% (P<0.05). Compared to the T1 and T2 treatments, the hemicellulose content of R2, R4, and R6 straw in the T3 treatment exhibited a significant decreasing trend (**Figure 3B**).

**Figure 4:**
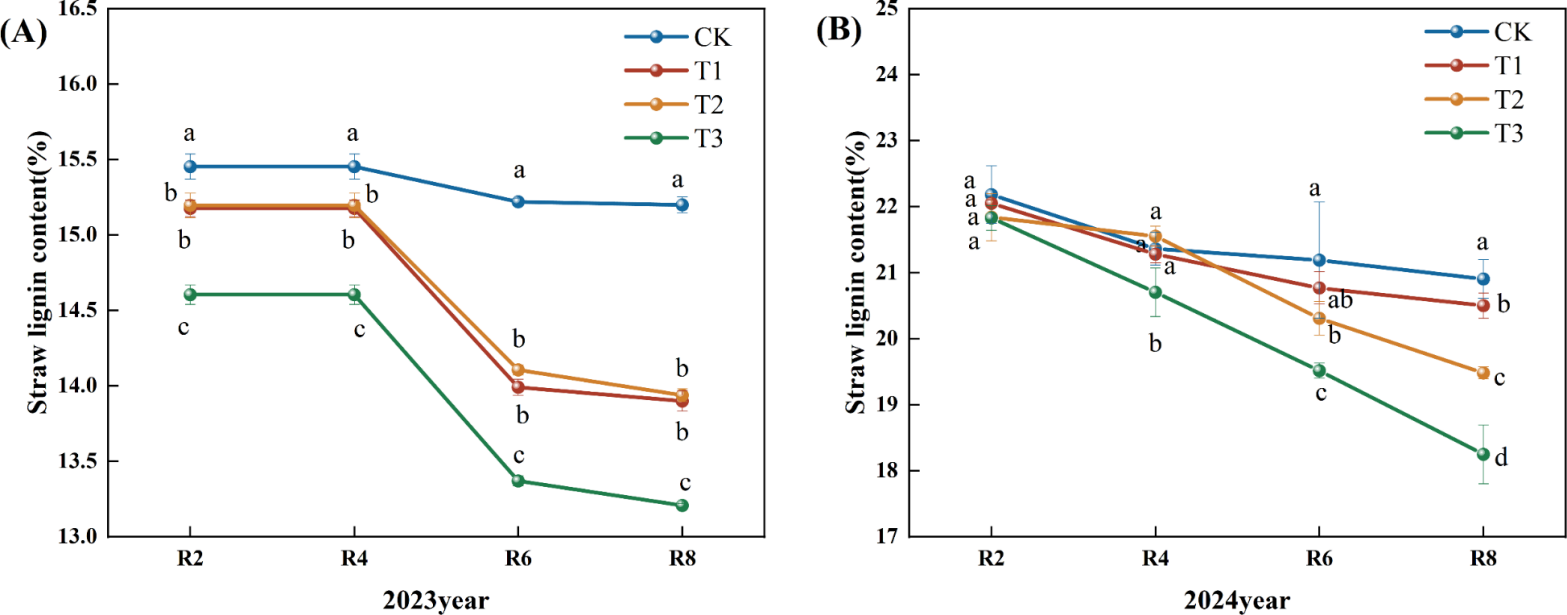
Effects of different microbial agents on lignin content of corn straw in two years. **(A)** The 2023 pot experiment. **(B)** The 2024 pot experiment. Different letters represent 5 % significant difference between treatments.

**Figure 4** illustrates a declining trend in straw lignin content over the growing period of 2023. In comparison to the CK, the straw lignin contents for treatments T1, T2, and T3 during the R2, R4, R6, and R8 periods exhibited significant reductions of 8.55%, 8.30%, and 13.11%, respectively (P<0.05). Furthermore, the T3 treatment demonstrated a significant decrease across all periods compared to the T1 and T2 treatments (**Figure 4A**). In 2024, the data indicated a declining trend in straw lignin content; relative to the CK, the T2 and T3 treatments significantly decreased straw lignin content during the R6 period by 2.00% and 4.00%, respectively (P<0.05). In the same comparison, the T1, T2, and T3 treatments significantly reduced straw lignin content during the R8 period by 7.00% and 13.00% (P<0.05). Compared to the T1 and T2 treatments, the T3 treatment exhibited a significant reduction at the R4, R6, and R8 periods (**Figure 4B**).

### 3.3 Effect of applying microbial inoculants on nutrient release rate of corn straw

**Figure 5:**
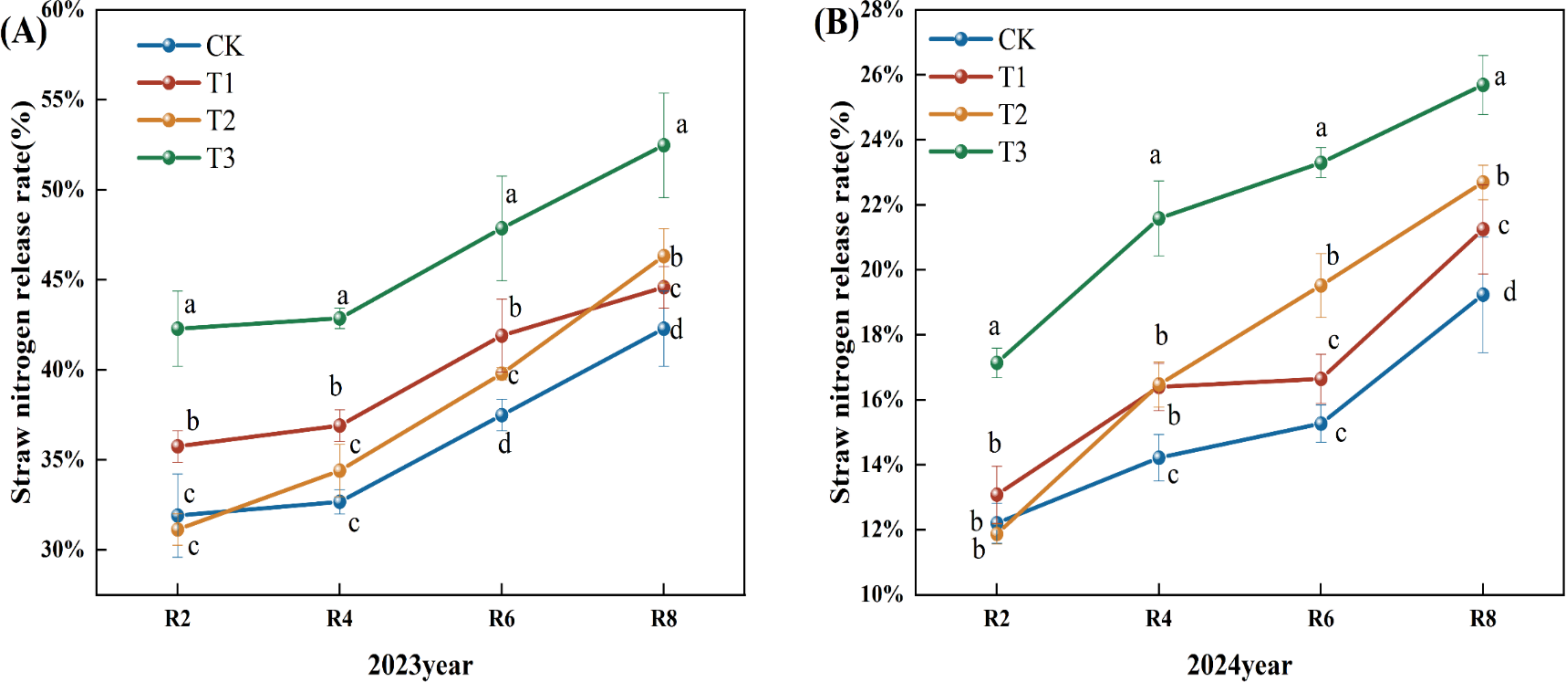
Effects of different microbial agents on nitrogen release rate of corn straw in two years. **(A)** The 2023 pot experiment. **(B)** The pot experiment in 2024. Different letters represent 5 % significant difference between treatments.

With the progress of the growth period, the nitrogen release rate of corn straw exhibited a generally increasing trend (**Figure 5**). In 2023, the nitrogen release rate from corn straw during the R2 to R4 period was slow, followed by an increase after R4. Compared to the CK treatment, the nitrogen release rates of straw in the T1, T2, and T3 treatments during the R4, R6, and R8 periods showed a significant increase. Specifically, during the R8 period, the T1, T2, and T3 treatments demonstrated increases of 5.46%, 9.55%, and 24.11%, respectively (P<0.05), compared to the CK treatment. Moreover, the T3 treatment exhibited a significant increase in the straw nitrogen release rate across all periods when compared to the T1 and T2 treatments (P< 0.05) (**Figure 5A**). In 2024, the nitrogen release rates for corn straw in the CK, T1, T2, and T3 treatments continued to increase. Relative to the CK treatment, the T1, T2, and T3 treatments significantly increased nitrogen release rates during the R4, R6, and R8 periods. At the R8 period, the increases for T1, T2, and T3 treatments were 10.00%, 18.00%, and 34.00%, respectively. Furthermore, the T3 treatment showed a significant increase in the straw nitrogen release rate at R2, R4, R6, and R8 stages compared to both T1 and T2 treatments (**Figure 5B**).

**Figure 6:**
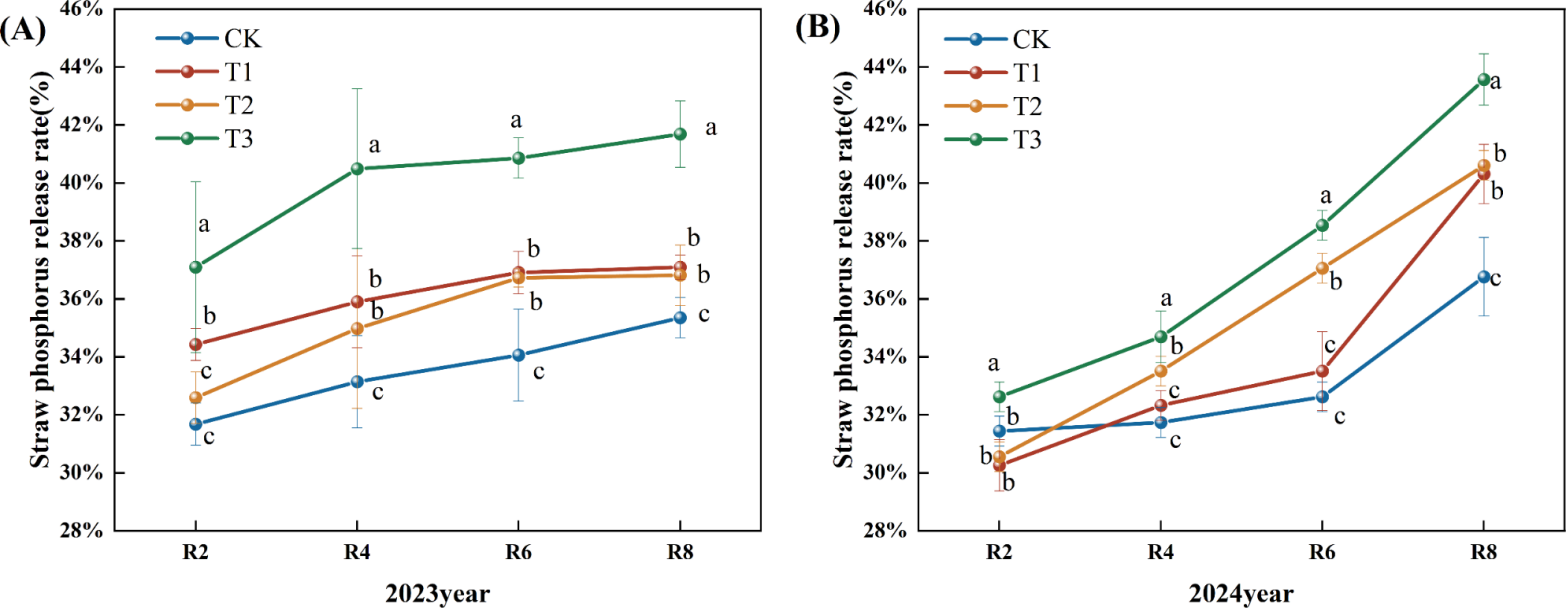
Effects of different microbial agents on phosphorus release rate of corn straw in two years. **(A)** The pot experiment in 2023. **(B)** The pot experiment in 2024. Different letters represent 5 % significant difference between treatments.

Throughout the growing period, the phosphorus release rate from maize straw exhibited a consistent upward trend (**Figure 6**). In 2023, the phosphorus release rate from corn straw escalated significantly from R2 to R4, then rose slower post-R4. In comparison to the CK, the straw phosphorus release rates for the T1, T2, and T3 treatments during the R2, R4, R6, and R8 periods exhibited a notable upward trend, with the release rates for the R8 period increasing significantly by 4.94%, 4.16%, and 17.93% for T1, T2, and T3, respectively (P<0.05). In comparison to the T1 and T2 treatments, the phosphorus release rate of straw at all stages of the T3 treatment exhibited a statistically significant increase (**Figure 6A**) (P<0.05). In 2024, the phosphorus release rate from corn straw steadily increased. Compared to the CK, the phosphorus release rate of straw at the R4, 6, and R8 periods exhibited a substantial upward trend. The phosphorus release rate from straw at the R8 period for treatments T1, T2, and T3 increased by 10.00%, 10.00%, and 19.00% (P<0.05). Compared to the T1 and T2 treatments, the phosphorus release rate of straw throughout each phase of the T3 treatment exhibited a substantial upward trend (**Figure 6**).

**Figure 7:**
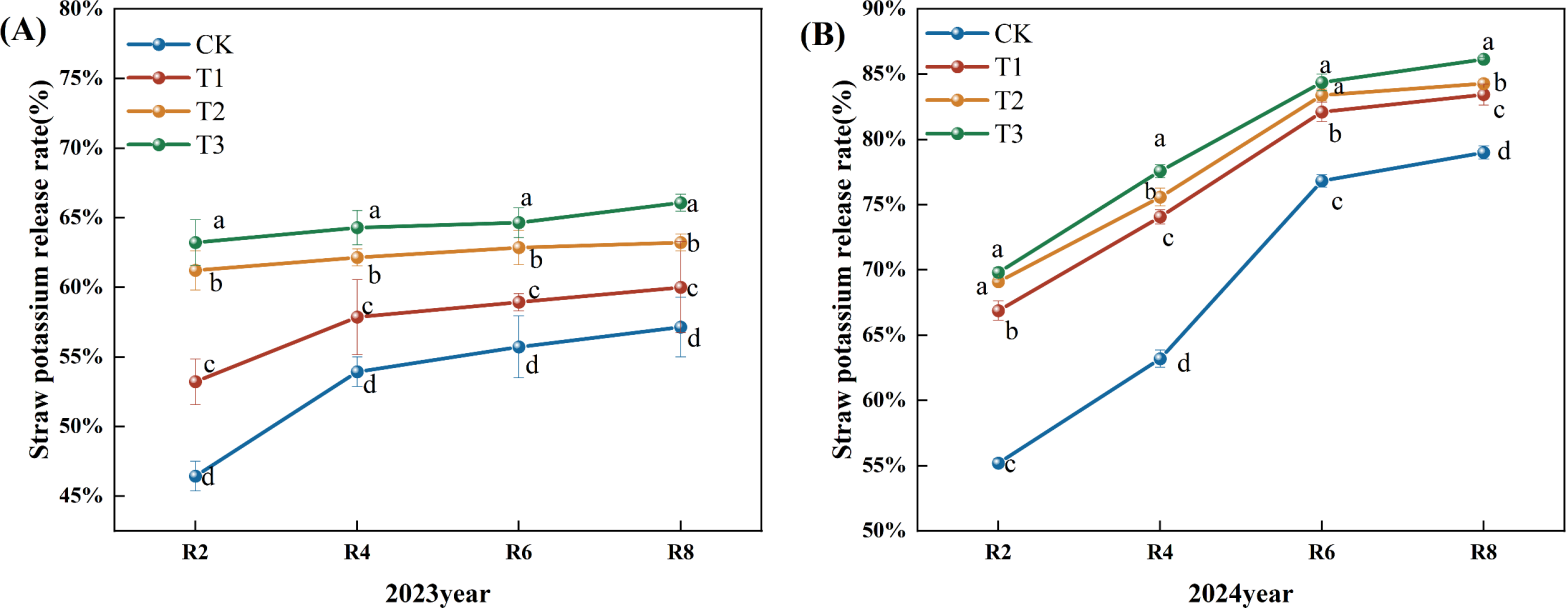
Effects of different microbial agents on potassium release rate of corn straw in two years. **(A)** The 2023 pot experiment. **(B)** The 2024 pot experiment. Different letters represent 5 % significant difference between treatments.

The release rate of potash from corn straw exhibited an upward trend as the fertility period progressed (**Figure 7**). In 2023, the release rate of potash from corn straw exhibited a gradual upward trend. In comparison to the CK, the straw potash release rates for the T1, T2, and T3 treatments during the R2, R4, R6, and R8 periods exhibited a notable upward trend, with the rates at the R8 period increasing significantly by 5.00%, 10.62%, and 15.62% (P<0.05). Furthermore, the straw potash release rates for the T3 treatment during all periods demonstrated a significant increase relative to the T1 and T2 treatments (**Figure 7A**). The potassium release rate from corn straw in 2024 exhibited a modest upward trend. In comparison to the CK, the rates of straw potash release during the R2, R4, R6, and R8 periods of the T1, T2, and T3 treatments were markedly elevated, with the R8 period exhibiting increases of 6.00%, 7.00%, and 9.00% for the T1, T2, and T3 treatments, respectively (P<0.05). Furthermore, the potassium release rate of straw in the R4 and R8 periods of the T3 treatment demonstrated a significant upward trend relative to the T1 and T2 treatments (**Figure 7B**).

### 3.4 Effect of applying microbial inoculants on the activity of extracellular enzymes in straw

**Figure 8:**
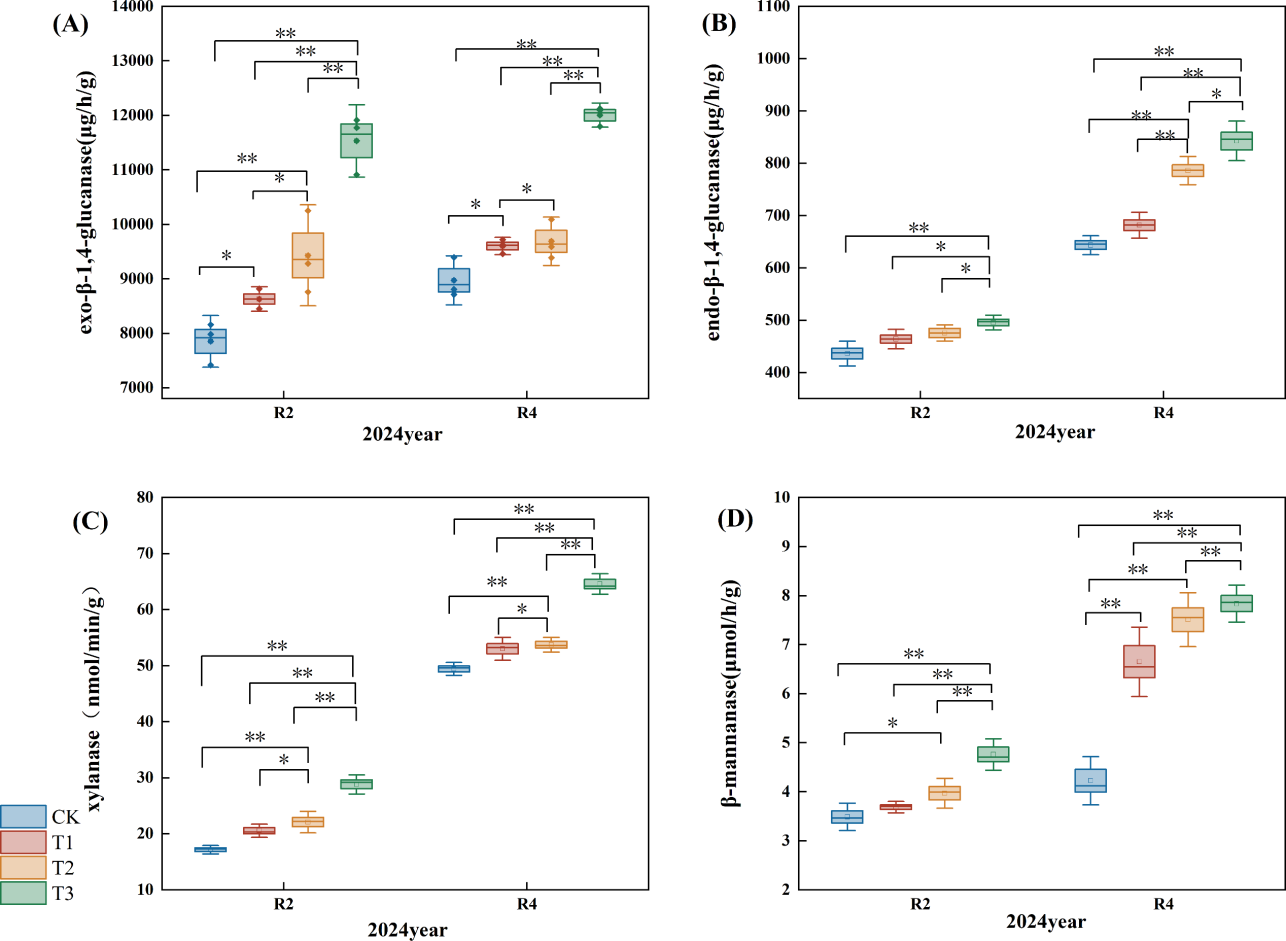
Effects of different microbial agents on the content of extracellular enzyme activity in straw. **(A)** exo-β-1,4-glucanase content in pot experiment. **(B)** endo-β-1,4-glucanase content in pot experiment. **(C)** xylanase content in pot experiment. **(D)** β-mannanase content in pot experiment. * and ** indicate that the correlation reaches the 0.05 and 0.01 significance levels, respectively.

Exo-β-1,4-glucanase primarily hydrolyzes the β-1,4-glycosidic bond sequentially from the non-reducing terminus of the cellulose polymer, yielding cellobiose or glucose. The exo-β-1,4-glucanase content exhibited an upward trend (**Figure 8A**); in the R2 period, the exo-β-1,4-glucanase levels for the T1, T2, and T3 treatments increased by 10.00%, 20.00%, and 47.00%, respectively, compared to the CK (P<0.05), with T3 demonstrating a highly significant difference from the T1 and T2 treatments. In the R4 period, the T1, T2, and T3 treatments elevated exo-β-1,4-glucanase content by 7.00%, 8.00%, and 34.00% compared to the CK (P<0.05), where T3 showed a highly significant difference from theT1 and T2, and T2 treatments exhibited a significant difference from the T1.

Endo-β-1,4-glucanase randomly cleaves β-1,4-glycosidic links in the cellulose chain, generating additional termini for exonuclease activity. The endo-β-1,4-glucanase content in straw exhibited an upward trend (**Figure 8B**); specifically, it increased by 6.00%, 9.00%, and 14.00% (P<0.05) in the T1, T2, and T3 treatments of R2 relative to the CK. Furthermore, the endo-β-1,4-glucanase content rose by 6.00%, 22.00%, and 31.00%, respectively (P<0.05), with a notable increase in the endo-β-1,4-glucanase content of straw in both T3 treatment R2 and R4 when compared to the T1 and T2 treatments.

Xylanase degrades xylan in hemicellulose, producing monosaccharides such as xylose. The xylanase content of straw exhibited an upward trend (**Figure 8C**); comparing with the CK, the xylanase levels in the R2 period for the T1, T2, and T3 treatments increased by 20.00%, 29.00%, and 68.00%, respectively (P<0.05). In the R4 period, the xylanase content for the T1, T2, and T3 treatments rose by 7.00%, 9.00%, and 31.00%, respectively (P<0.05). Notably, the xylanase content at the R2 and R4 periods of the T3 treatment demonstrated a significant increase compared to the T1 and T2 treatments.

β-annanase catalyzes the degradation of mannans in hemicellulose, resulting in the liberation of mannose. The β-mannanase concentration within straw exhibited an upward trend (**Figure 8D**); during the R2 period, the β-mannanase levels in the T1, T2, and T3 treatments rose by 5.00%, 14.00%, and 36.00%, respectively, compared to the CK (P<0.05); in the R4 period, the increases for the T1, T2, and T3 treatments were 57.00%, 78.00%, and 85.00%, respectively (P<0.05); additionally, a notable enhancement in straw xylanase content was recorded in the T3 treatment at both R2 and R4 periods when compared to the T1 and T2 treatments.

### 3.5 Effect of applying microbial inoculants on soybean yield and its components

As shown in **Table 3**, the pod number per plant of soybean in the T3 treatment was significantly higher than that in the CK, T1 and T2 treatments, and the value was the largest in 2023; the grain number per plant,100-grain weight and grain weight per plant in the T2, and T3 treatments were significantly higher than those in the CK and T1 treatments; when compared with that in the CK, the increase in grain weight per plant in theT1, T2 and T3 treatments was 15.00%, 23.00% and 28.00% (P<0.05). In 2024, the grain number per plant in the T3 treatment was significantly different from the CK, T1, and T2 treatments; there was no significant difference in the content of 100-grain weight among the CK, T1, T2 and T3 treatments; the pod number per plant in the T2 and T3 treatments was significantly higher than that of the CK and T1 treatments; and the grain number per plant in the T1, T2 and T3 treatments increased by 4.00%, 7.00% and 24.00% (P<0.05) compared with the CK. In conclusion, T3 treatment had a better promotion effect on grain weight per plant.

**Table 3:** Effects of different preservatives on soybean yield and its components.

year	Treatment	Weight per plant	100-grain weight	Pod number per plant	Theoretical yield
2023	CK	43.44 ± 2.01c	18.81 ± 0.42b	23.11 ± 0.51b	8.24 ± 0.12c
	T1	49.67 ± 3.00b	19.07 ± 0.57ab	23.44 ± 2.69b	9.47 ± 0.37b
	T2	52.67 ± 2.96a	19.30 ± 0.3ab	24.56 ± 1.84b	10.17 ± 0.2a
	T3	54.11 ± 3.01a	19.44 ± 0.38a	29.11 ± 1.17a	10.54 ± 0.32a
2024	CK	38.22 ± 2.83c	18.05 ± 0.26a	17.06 ± 0.52c	6.90 ± 0.47b
	T1	39.56 ± 3.15bc	18.19 ± 0.56a	19.74 ± 0.87b	7.20 ± 0.24b
	T2	40.44 ± 2.92b	18.21 ± 0.51a	22.39 ± 0.42a	7.36 ± 0.56b
	T3	46.69 ± 3.03a	18.37 ± 0.22a	22.41 ± 0.5a	8.58 ± 0.66a

Different lowercase letters indicate significant differences between treatments (*p* < 0.05).

### 3.6 Scanning electron microscopy analysis

**Figure 9:**
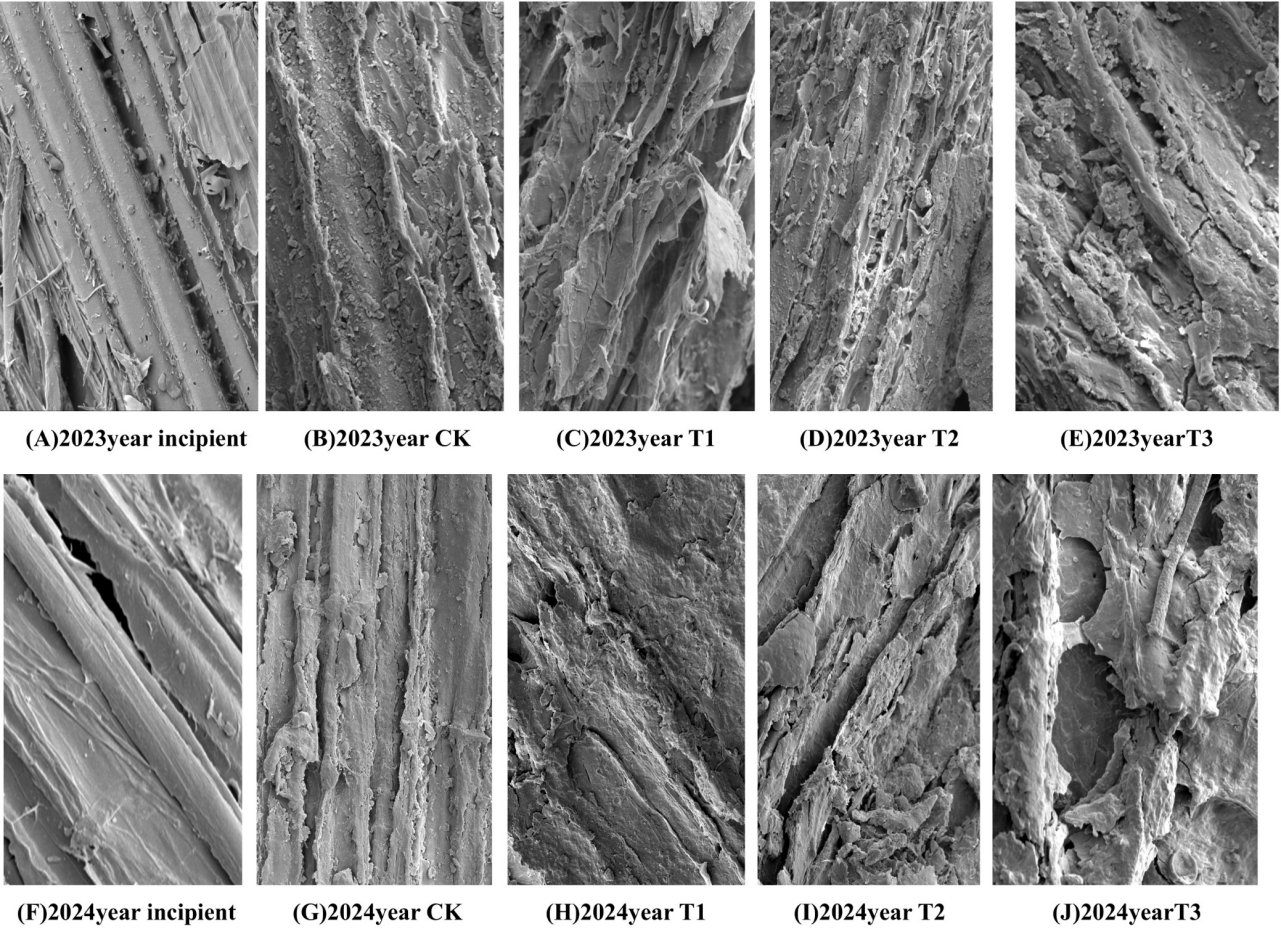
Scanning electron microscopy of straw surface structure at maturity stage in two years. **(A–E)** Electron microscope scans of each treatment after the addition of microbial agents in 2023. **(F–J)** Electron microscope scans of each treatment after the addition of microbial agents in 2024.

As can be seen in **Figure 9**, scanning electron microscope observation showed that the surface of the initial straw (**Figures 9A, F**) was smooth, regular, and structurally intact, and the epidermal structure covered the internal lignocellulose; the lignocellulose was structurally regular, flat, and smooth, and the surface was aligned neatly. After the straw was returned to the field and buried, the surface structure of the straw in each treatment was analyzed by scanning electron microscope at the stage of soybean R8 period. Compared with the initial straw, the surface of the later one became rough. Larger hollows appeared on the surface of the straw with the epidermal tissues under its waxy-silicified layer gradually revealed, which led to the destruction of the original cell wall and the complex fiber structure, and the straw showed a fractured shape and uneven faults, so many pores of different sizes appeared on the surface. Compared with the CK (**Figures 9B, G**), T1 (**Figures 9C, H**), and T2 (**Figures 9D, I**) treatments, the T3 treatment (**Figures 9E, J**) treated corn straw had a high degree of non-uniformity in appearance, an increase in the number of voids and cracks on the surface of the straw, a high degree of crushing and looseness, the appearance of finely chopped scales. In other words, the surface of the straw showed a severely eroded appearance, exposing more internal voids.

### 3.7 Partial least squares path model of each index after adding different microbial inoculants

**Figure 10:**
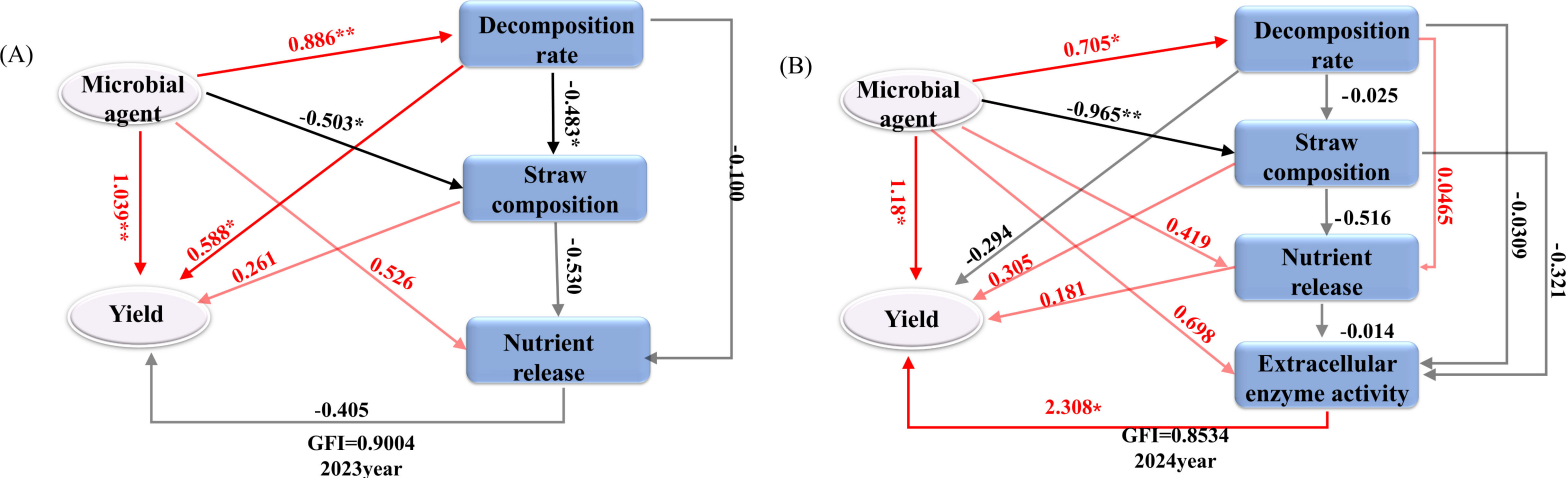
Partial least squares path model structural equation model that affects each index after adding different bacteria. **(A)** 2023 Potting experiment. **(B)** 2023 Potting experiment. Note: Numbers next to the arrows indicate path coefficients, with red indicating a positive effect and black indicating a negative effect. * and ** indicate that the correlation reaches the 0.05 and 0.01 significance levels, respectively.

**Figure 10** illustrates that partial least squares (PLS) were employed to develop path models assessing the impact of various microbial inoculants and indicators on straw decomposition rate, straw composition, nutrient release, extracellular enzyme activity, and theoretical soybean yield (grain weight per plant) in 2023 and 2024. The goodness-of-fit indices (GFI) for the models were 0.9004 (2023) and 0.8534 (2024). The response variables included the addition of different microbial agents, straw decomposition rate, straw composition (cellulose, hemicellulose, and lignin), nutrient release (N, P, and K), and extracellular enzyme activities (2024) such as exo-β-1,4-glucanase, endo-β-1,4-glucanase, xylanase, and β-mannanase. The influence of various microbial agents on straw breakdown rate, individual plant grain weight, and straw lignocellulose content was more pronounced following the application of different microbial agents in 2023, with path coefficients of 0.886, 1.039, and -0.503, respectively, indicating a significant level of impact. The impact of straw breakdown rate on individual plant grain weight and lignocellulose content was significant, with path coefficients of 0.588 and -0.483, respectively, indicating a notable level of influence. In 2024, several microbial agents significantly influenced the straw breakdown rate, the number of grains per plant, and straw composition, with path coefficients of 0.705, 1.093, and -0.965, respectively. All attained a notable level. The activity of straw extracellular enzymes predominantly influenced the grain weight of individual soybean plants. The path coefficient was 2.308, which was statistically significant.

### 3.8 Mantel-test correlation heat map analysis of each index after adding different microbial inoculants

**Figure 11:**
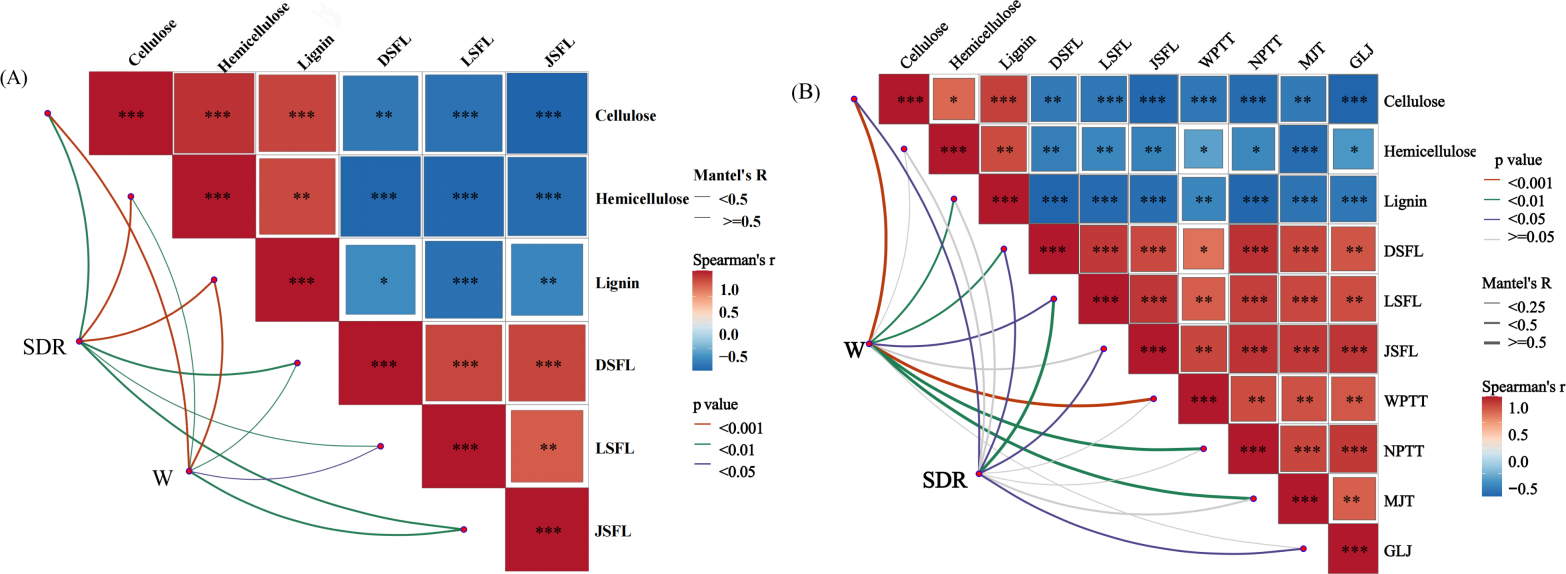
Mantel-test correlation analysis heat map of each index after adding different microbial agents. **(A)** In 2023, the Mantel-test correlation analysis heat map of each indicator after different treatments will be added. **(B)** In 2024, the Mantel-test correlation analysis heat map of each indicator after different treatments will be added. SDR, straw decomposition rate; W, single plant weight; DSFL, straw nitrogen release rate; LSFL, straw phosphorus release rate; JDFL, straw potassium release rate; WPTT, exo-β-1,4-glucanase; NPTT, endo-β-1,4-glucanase; MJT, xylanase; GLT, β-mannanase. *,**and *** indicate that the correlation reaches the 0.05 , 0.01 and 0.001 significance levels, respectively.

As can be seen from **Figure 11**, the Mantel-test correlation heat map in 2023 indicated that significant differences were reached between straw decomposition rate and cellulose, hemicellulose, lignin, nitrogen, phosphorus, and potassium release rates after the addition of different microbial agents (P<0.05). By contrast, significant differences were reached between single-plant grain weight and cellulose, hemicellulose, lignin, nitrogen release rate, and potassium release rate after adding different microbial agents (P<0.05). The correlation heatmap 2024 showed certain differences (P<0.05) between grain weight and cellulose, lignin, nitrogen release rate from straw, exo-β-1,4-glucanase, endo-β-1,4-glucanase, xylanase. In short, after adding different microbial agents, the straw decomposition and phosphorus release rates reached significant differences.

## 4 Discussion

The research results of 2 consecutive years showed that straw decomposition was characterized by fast in the early stage and slow in the late stage. The rapid decomposition period was mainly concentrated in the R2-R4 periods. This stage is the key period for releasing mineral nutrients due to the rapid decomposition of easy-to-mineralize organic matter in straw. After the R4 period, it was the time of slow straw decomposition. The difficult-to-decompose materials in straw (such as lignin, waxes, tannins, etc.) were gradually degraded by fungi and actinomycetes (Yao et al., 2020). In the R8 period, the cumulative decomposition rate of corn straw ranged from 62.00% to 87.56%. The T1, T2, and T3 treatments exhibited a faster decomposition rate than the CK. This enhanced degradation is attributable to the microbial agents’ ability to facilitate the breakdown of the substances which are more difficult to degrade in the straw (Voberkova et al., 2017; Zhang et al., 2022). Notably, the decomposition rate of the T3 treatment was significantly higher than that of the T1 and T2 treatments because of the synergistic effect produced by the compounding microbial agents. Furthermore, the compounded microbial agents could degrade different difficult-to-decompose substances in straw more comprehensively, and the T3 treatments may have complemented each other’s enzyme systems during the degradation process, enhancing the overall degradation efficiency. Specifically, the synergistic action of these enzymes is a key mechanism for the efficient degradation of lignocellulose. And the synergistic action of endoglucanase and exoglucanase, combined with further decomposition by β-glucosidase and xylanase, can convert complex lignocellulosic structures into utilizable monosaccharides. Inoculation of lignocellulosic-degrading microorganisms provides a promising solution for accelerating lignocellulosic degradation. The effectiveness of inoculation with a single microbial strain to promote lignocellulose degradation was unstable. At the meantime, the inoculation of a synthetic community consisting of multiple lignocellulose-degrading fungal or bacterial taxa was more effective than that of a single strain (Liu Q. M., et al., 2025). In addition, the composite microbial agents may have played a more balanced role in the different stages of straw decomposition, accelerating the degradation of labile substances during the rapid decomposition period of straw, thus significantly increasing the overall decomposition rate. While at the R8 stage, the straw decomposition rate of treatments with applying microbial agents (T1, T2, and T3) was not significant with the CK, the chemical structure of mature straw is more complex, making it difficult for microorganisms to decompose further. This limitation of substrate availability may be responsible for the insignificant differences in decomposition rates (Liu D et al. 2025); the regulation of C/N by applying additional exogenous nitrogen only had a specific promotional effect on the early stage of corn straw decomposition. The final straw decomposition rate of the microbial agent application was not significantly different from the reference, similar to the previous study (Zhang J. T. et al., 2018). Straw transformation in soil is a complex biochemical process in which microorganisms, as a key driver, dominate the transformation of organic matter and nutrient cycling. Numerous studies have been focused on the correlation between functional microorganisms and organic matter decomposition (Voberkova et al., 2017; Wang and Fan, 2010). It has been indicated that the addition of straw decomposition microbial agents can significantly increase the number of microorganisms associated with straw decomposition and enhance their metabolic activities under suitable environmental conditions, which in turn enhances the activities of key enzymes (e.g., cellul0se, hemicellulose, and lignin), to ultimately facilitate the efficient decomposition of straw and the release of nutrients (Wei et al., 2012).

Compared with CK, T1, and T2 treatments, the decreasing rate of cellulose, hemicellulose, and lignin content in the T3 treatment was significantly higher than that in other treatments, which might be due to the fact that the strain in the T3 treatment could secrete some secondary metabolites faster, improving the microenvironment of the straw and promoting the synergistic effect of other microorganisms; the reason for the slower rate of decomposition at the later stage might be that exogenous nitrogen inhibited the decomposition of lignin and other difficult-to-degrade materials with the advancement of the degradation process of the straw, thus the decomposition rate of straw slowed down (Zhang J. T. et al., 2018). Similar conclusions were reached by Zeng Li (Zeng et al., 2020) in a study on the effects of different nitrogen application rates on wheat straw decomposition promotion. Exogenous nitrogen addition has been shown to significantly increase the activity of hydrolytic enzymes associated with the decomposition of carbohydrates such as cellulose. However, simultaneously, it inhibited the activity of oxidative enzymes associated with lignin degradation (Sinsabaugh et al., 2002).

With the progress of straw decomposition and straw decomposition, the exo-β-1,4-glucanase, exo-β-1,4-glucanase, xylanase, and β-mannanase of straw showed the same increasing trend. However, the T3 treatment performed best, significantly higher than the CK and T1 and T2 treatments. Thus, it had an important role in the putrefaction of straw; the reason for the faster increase in the T3 treatment may be that the application of composite microbial agent decomposed more of the complex polysaccharide structure in straw, producing more substrate fragments, which efficiently to act in the enzymatic reaction; the faster conversion of large molecular sugars into small molecular sugars, which ultimately manifested itself in the significant increase in the enzyme activity. The straw decomposition process relies on the soil’s microbial community to break down the straw and convert it into organic matter. However, the enzyme system secreted by a single microbial community is limited, which makes it challenging to achieve complete straw degradation. To overcome this obstacle, it is necessary to employ multiple microorganisms because they can produce multiple enzyme systems. That’s how to facilitate a synergistic degradation process (Wang et al. 2025). In conclusion, the correlation analysis of this study revealed that exo-β-1,4-glucanase, exo-β-1,4-glucanase, xylanase, and β-mannanase were necessary factors affecting the nutrient release from straw.

The structure of corn straw mainly consists of the epidermis, mechanical tissue, vascular bundles, and essential tissue. Its decomposition is mainly due to the destruction of the thin-walled cells of the essential tissue and the best in the vascular bundles (Makri et al., 2024). Scanning electron microscopy (SEM) can visually reflect the changes in the external structure of straw and the degree of damage during the decomposition process. It was found that the number of voids in the straw residues increased with the prolongation of the decomposition time, which was mainly rooted in the decomposition of a large number of carbohydrates (including hemicellulose and cellulose). However, lignin was more complex in structure and more challenging to decompose than other carbohydrates. The destruction degree of straw in the microbial agent treatment was significantly greater than that in the non-nitrogen treatment, further indicating that the application of decomposing agent can promote the decomposition of corn straw, which is in line with the previous study (Zeng et al., 2020).

Straw contains many nitrogen, phosphorus, potassium, and various micronutrients. Therefore, the process of straw decomposition is accompanied by the release of straw nutrients. The present study found that the dynamics of straw decomposition and nutrient release also showed a trend from fast to slow under CK, T1, T2, and T3 treatments. In the meantime, the difference between the straw decomposition rate of T1, T2, and T3 treatments and that of the CK was gradually reduced with the prolongation of the decomposition time. The result agrees with the study conclusion conducted by the previous study (Zhao et al., 2024). In terms of the magnitude of the nitrogen, phosphorus, and potassium nutrient release rates at the experiment's end, the potassium release rate was the largest and basically completed at the R6 period. The reason is that the potassium in the straw mainly existed in the water-soluble state, which was readily soluble in water and released rapidly. The release rate of phosphorus was increasing because more than 60% of phosphorus in straw existed in ionic form, and the remaining part was mainly involved in the composition of the cell wall. Then, the inorganic phosphorus, easily soluble in water, was gradually released in the early stage, while the organic phosphorus was more challenging to generate. In other words, the phosphorus release rate from straw increased slowly in the later stage, consistent with the results of the previous work (Zeng et al., 2020).

Nitrogen in straw can be roughly divided into storage nitrogen and structural nitrogen. Storage nitrogen includes nitrate nitrogen (NO3--N), ammonium nitrogen (NH4+-N), and some small molecules of organic nitrogen (amino acids, amides, etc.); structural nitrogen is mainly organic nitrogen that is refractory to decompose, involving chlorophyll, proteins (enzymes), nucleic acids, amines, amine compounds, and a variety of vitamins in the nitrogen. Storage nitrogen is quickly released from straw, but the proportion is very low; structural nitrogen is the main component of straw nitrogen, which must first be mineralized by microorganisms into inorganic nitrogen to be gradually released, and the process is relatively slow. Therefore, the rate of nutrient release from straw was manifested as K>P>N (Zhang J. T. et al., 2018). This 2023 study reveals a distinct finding: the nutrient release rate from straw is K>N>P, which contradicts earlier research. This discrepancy may be attributed to phosphorus predominantly existing as organic phosphorus and insoluble inorganic phosphorus. The release of phosphorus is hindered by soil fixation and mineralization processes, restricting its mobility and efficacy, resulting in a slower release rate that may be inferior to that of nitrogen (Li H.D. et al., 2024).

Applying microbial inoculants by incorporating straw into the soil can enhance crop yield. According to Xiao et al. (2023), applying microbial inoculants could significantly increase the yield, and the 45 kg/hm2 microbial inoculant dosage treatment increased by 11.59% compared with the reference. Zhang S. Y. et al. (2018) concluded that incorporating straw into the soil and incorporating straw into the soil combined with microbial agents had significant yield increase effects on rice, with the yield increased by 6.40% and 10.40%, respectively. This study found that the increase in per grain weight of T3 treatment was 24.00%-28.00% at the R8 stage. Compared with CK, T1, and T2 treatments, the T3 treatment had a better promotion effect on the grain weight of a single plant, consistent with the previous study. This proved that there was an effect of yield increase after the application of microbial inoculants. This study found that Bacillus was able to promote the decomposition of straw and the release of nutrients, which may be the reason for the increase in crop yield.

Scanning electron microscopy (SEM) analysis provides important evidence for intuitively demonstrating the effects of microbial inoculant addition on straw decomposition (**Figure 9**). These changes in microstructure reflect the promoting effect of inoculant addition on straw decomposition. The surface of straw treated with T3 (a composite inoculant) shows more cavities and cracks, indicating that the microorganisms in the inoculant can effectively penetrate the epidermal structure of the straw, accelerating the degradation of lignocellulose. This degradation not only alters the physical structure of the straw but may also enhance the availability of its chemical components, thereby facilitating the release of nutrients. Moreover, the more significant fragmentation and looseness of the surface of straw treated with T3 suggest that adding inoculants may enhance microbial synergism, further accelerating the decomposition process of the straw. The results of SEM analysis provide intuitive evidence of the effects of inoculant addition on straw decomposition, indicating that inoculant application can significantly alter the microstructure of straw and accelerate its decomposition. This finding offers important theoretical support for optimizing inoculant application and improving the effectiveness of straw incorporation.

As the Partial least squares path model reveals that in 2023, adding different microbial inoculants significantly enhanced the decomposition rate of straw (**Figure 10**). The inoculants also had a significant positive impact on the weight of individual soybean plants while exerting a significant negative influence on the lignocellulose content. This indicates that the inoculants accelerated straw decomposition, reduced the residual lignocellulose in the straw, and thereby improved the efficiency of nutrient release. In 2024, the impact of inoculant addition on the decomposition rate of straw and the number of individual plants was even more pronounced. The decomposition rate of straw, the rate of nutrient release, and the activity of extracellular enzymes are key factors affecting crop yield. Optimizing the addition of microbial inoculants and management practices can significantly enhance the efficiency of straw decomposition and the rate of nutrient release, thereby promoting crop growth and yield. These findings provide important theoretical support for optimizing straw incorporation and the application of microbial inoculants.

Compared with the CK, the rate of applying microbial agents in straw decomposition showed a significant increase, a gradual decrease trend in hemicellulose content, a gradual increase in nitrogen, phosphorus, and potassium release from straw, a gradual increase in extracellular enzyme activity and the soybean yield. Compared with T1 and T2, T3 treatment increased the content of straw exo-β-1,4-glucanase and straw xylanase in the R2 and R4 periods. As the scanning electron microscope image showed, the cavities and cracks on the straw surface increase, break loosely, and show severe erosion; the hemicellulose of the straw significantly decreased in the R4 and R6 periods; and the decomposition rate of the straw significantly increased in the R8 period. In summary, T3 treatment had the best effect in promoting straw decomposition, reducing the content of straw cellulose, hemicellulose, and lignin, increasing the activity of extracellular enzymes, accelerating the degree of straw surface fragmentation, etc. It can provide the theoretical basis and technical support for the promotion of incorporating straw into the soil and decomposition in saline and alkaline.

## 5 Conclusion

Compared with the CK treatment, the rate of applying microbial agents in straw decomposition showed a significant increase; a gradual decreasing trend in hemicellulose content; a gradual increase in nitrogen, phosphorus, and potassium release from straw; and a gradual increase in extracellular enzyme activity and the soybean yield. Compared with T1 and T2, the T3 treatment increased the content of straw exo-β-1,4-glucanase and straw xylanase in the R2 and R4 periods. As the scanning electron microscopy image showed, the cavities and cracks on the straw surface increased, broke loosely, and showed serious erosion; the hemicellulose of the straw significantly decreased in the R4 and R6 periods; the decomposition rate of the straw significantly increased in the R8 period. In summary, the T3 treatment had the best effect in promoting straw decomposition; reducing the content of straw cellulose, hemicellulose, and lignin; increasing the activity of extracellular enzymes; and accelerating the degree of straw surface fragmentation, etc. It can provide the theoretical basis and technical support for the promotion of incorporating straw into the soil and decomposition in saline and alkaline.

## Data Availability

The datasets presented in this study can be found in online repositories. The names of the repository/repositories and accession number(s) can be found in the article/**Supplementary Material**.
