# Supplementary material for: Effects of rot-promoting bacteria on decomposition characteristics of corn straw and spring soybean yield in Saline-alkali Land
Source: Front Plant Sci. 2025 May 13;16:1572868. doi: 10.3389/fpls.2025.1572868 (PMC12106462; doi:10.3389/fpls.2025.1572868)
Supplement: Supplementary file 1 [file DataSheet1.zip › Supplementary_Figure.docx]

Supplementary Material

# Supplementary Figures and Tables

## Supplementary Figures


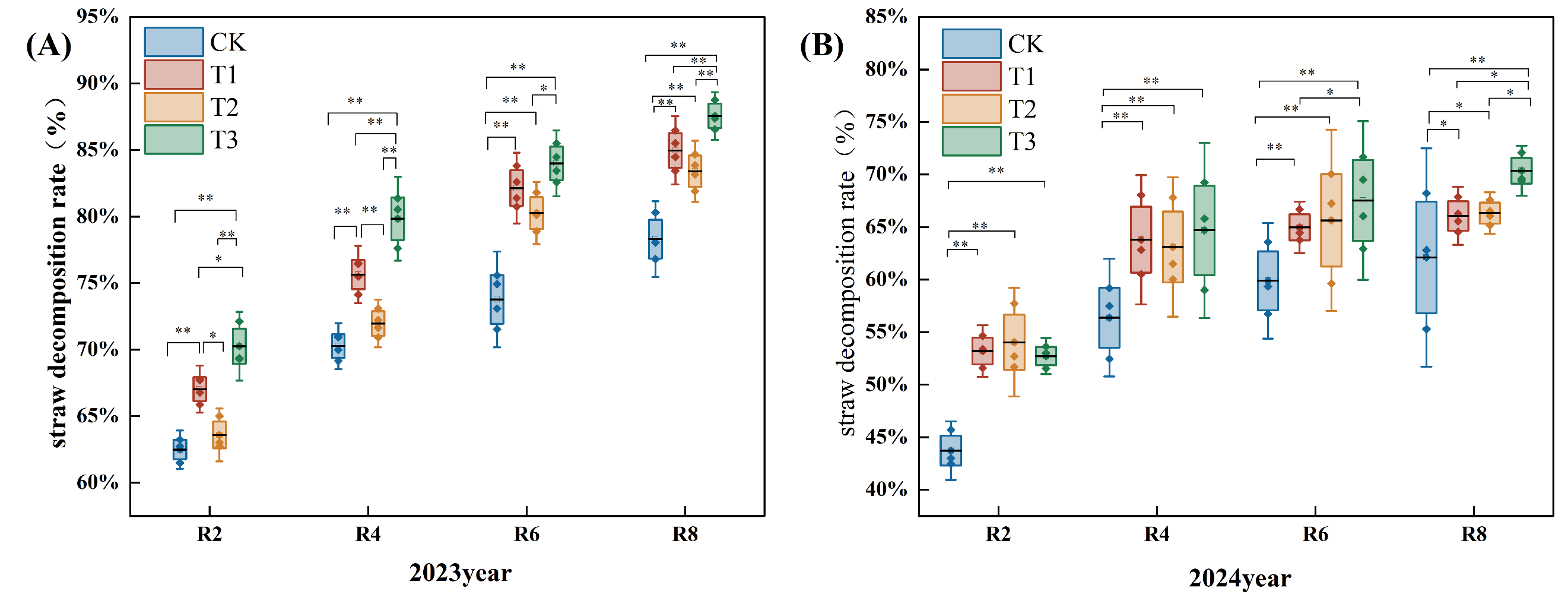


**Figure 1.** The effect of different exogenous microbial agents on the decomposition rate of straw in two years.

**
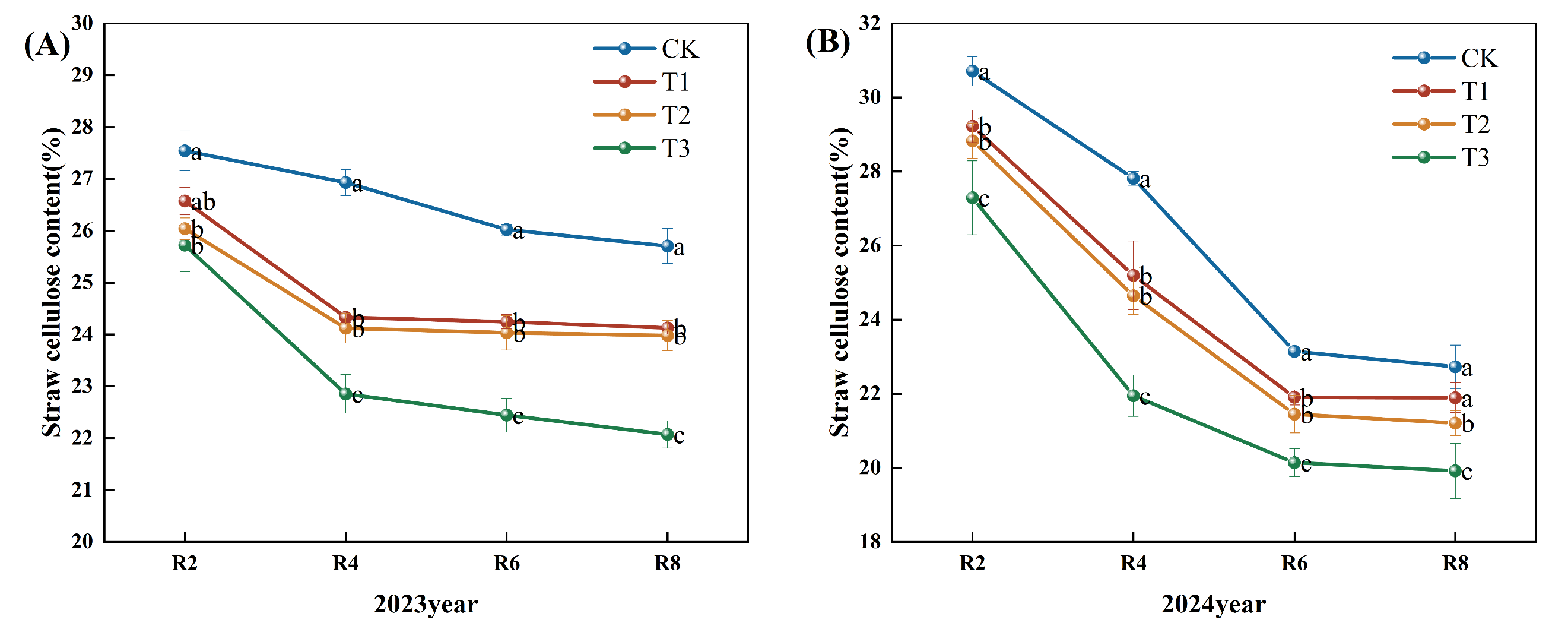
**

**Figure 2.** Effects of different microbial agents on cellulose content of corn straw in two years.


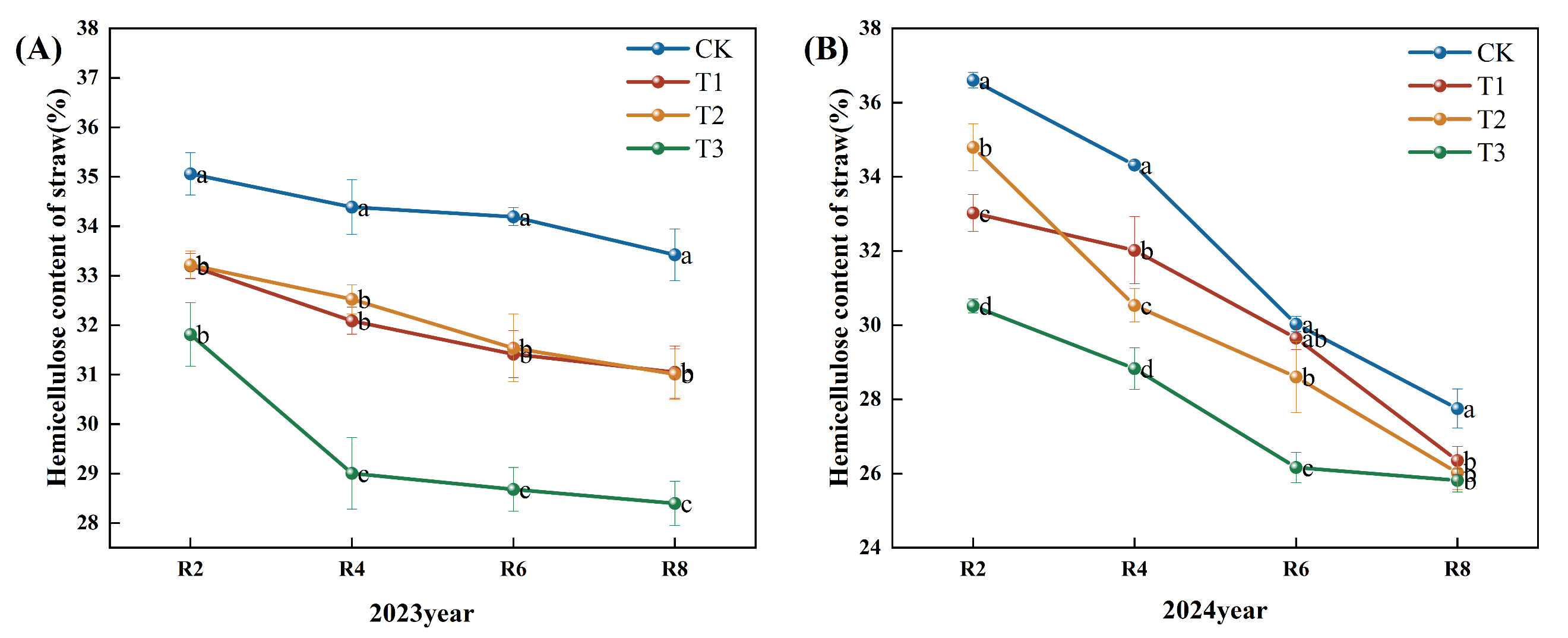


**Figure 3.** Effects of different microbial agents on hemicellulose content of corn straw in two years.


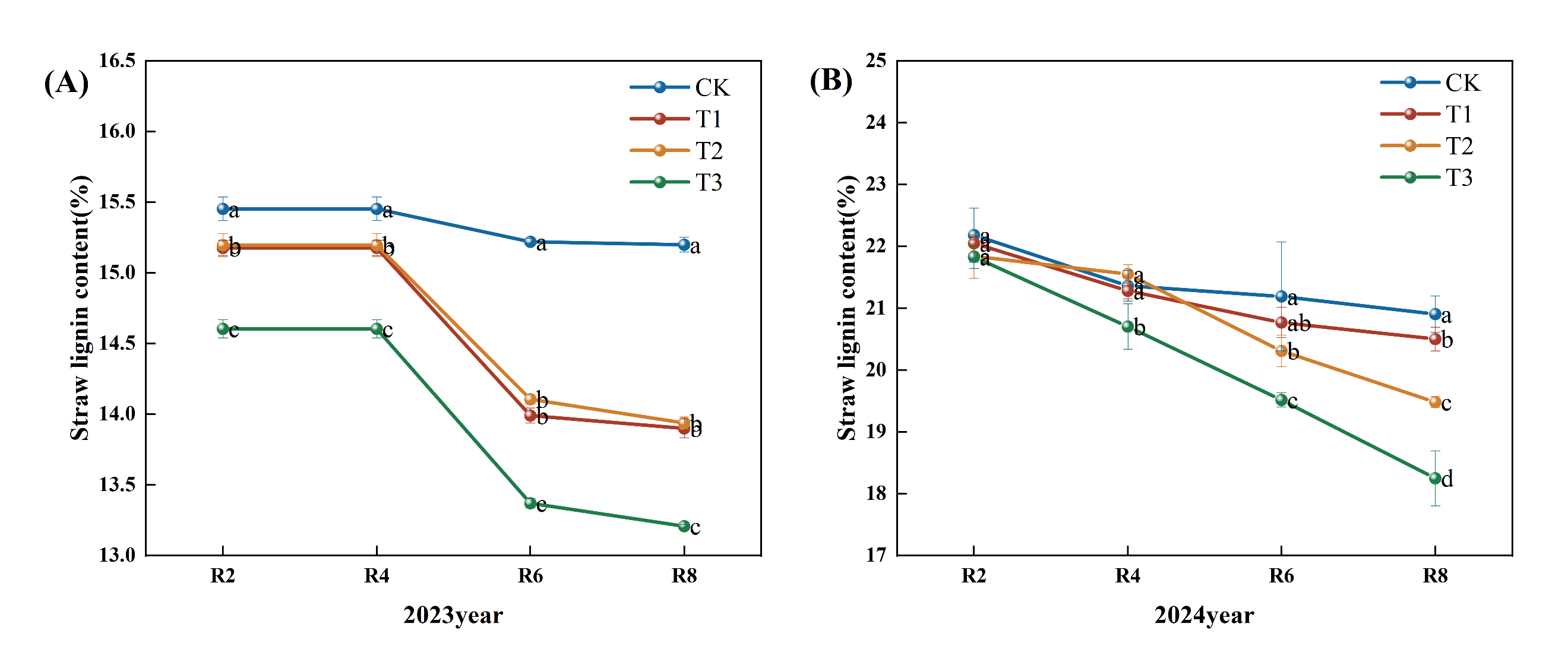


**Figure 4.** Effects of different microbial agents on lignin content of corn straw in two years.


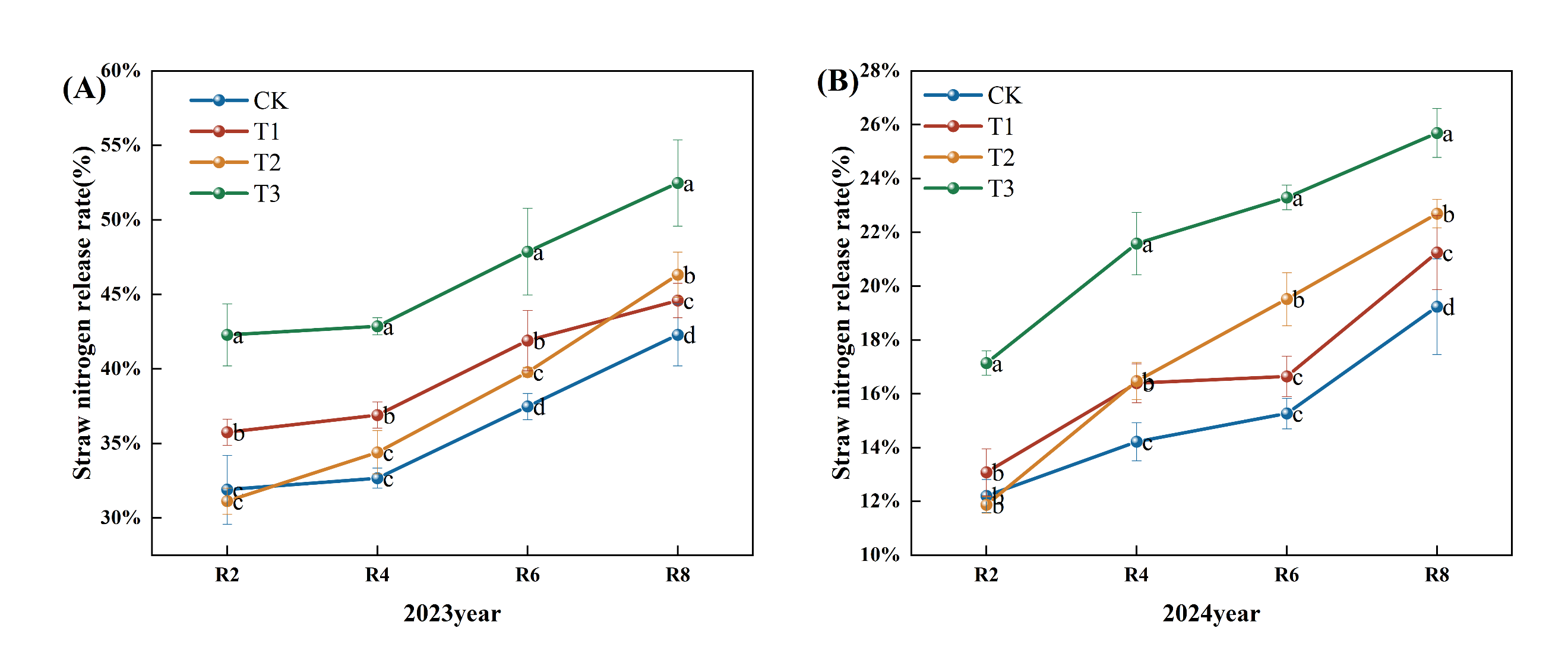


**Figure 5.** Effects of different microbial agents on nitrogen release rate of maize straw in two years.


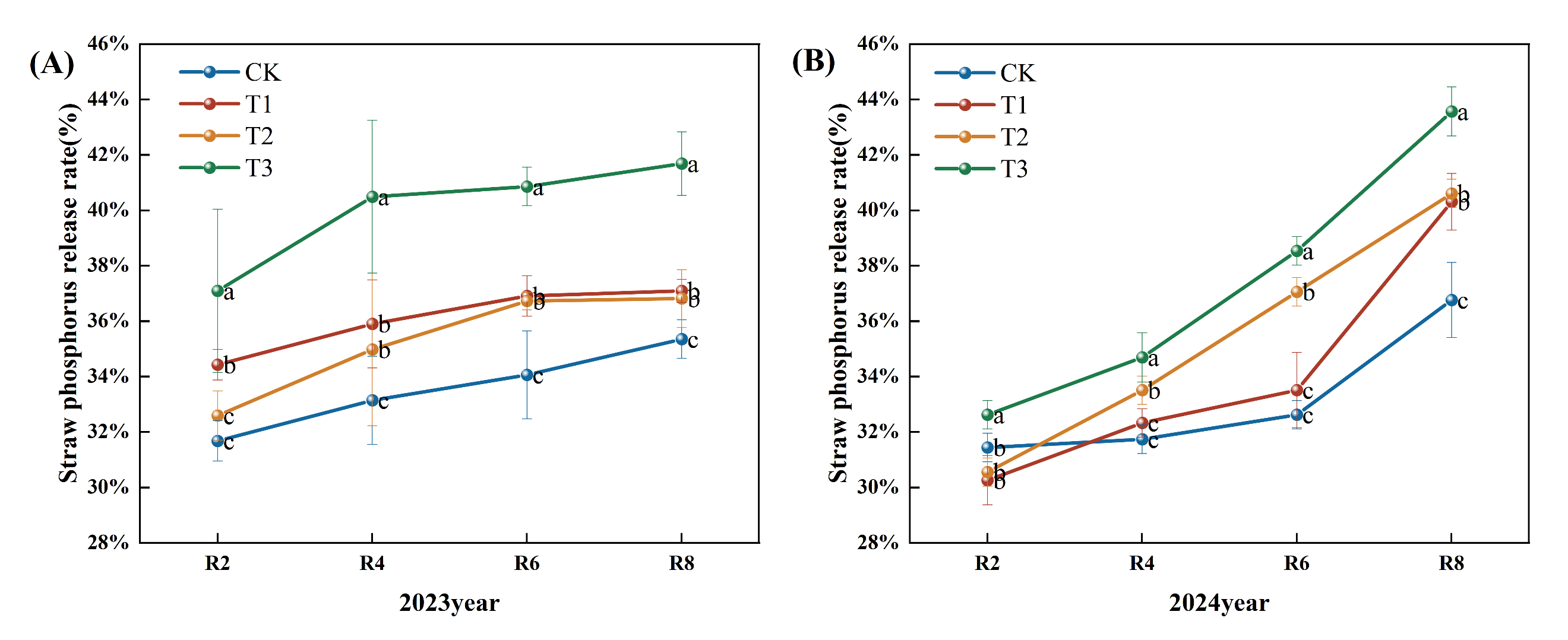


**Figure 6.** Effects of different microbial agents on phosphorus release rate of corn straw in two years.


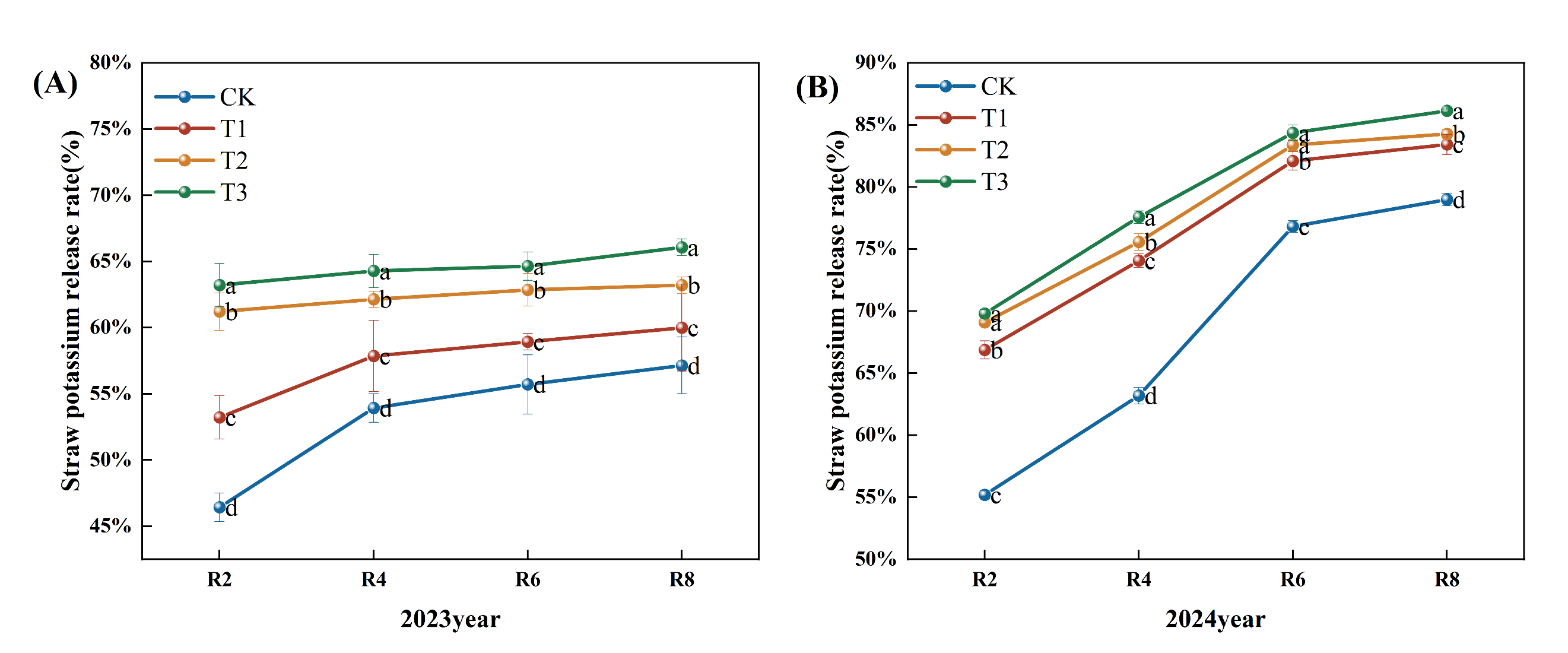


**Figure 7.** Effects of different microbial agents on potassium release rate of corn straw in two years.


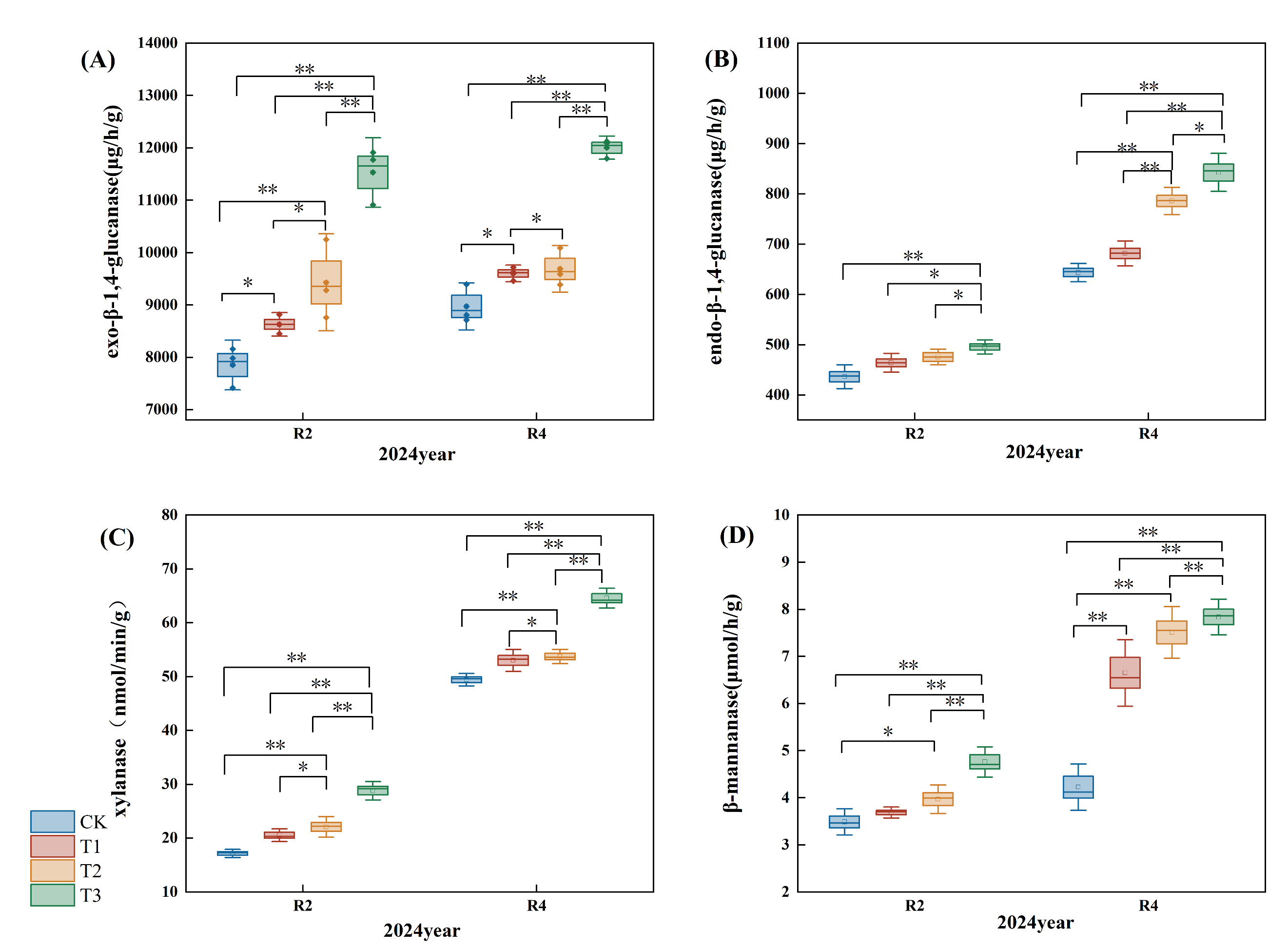


**Figure 8.** Effects of different microbial agents on the content of extracellular enzyme activity in straw.

| 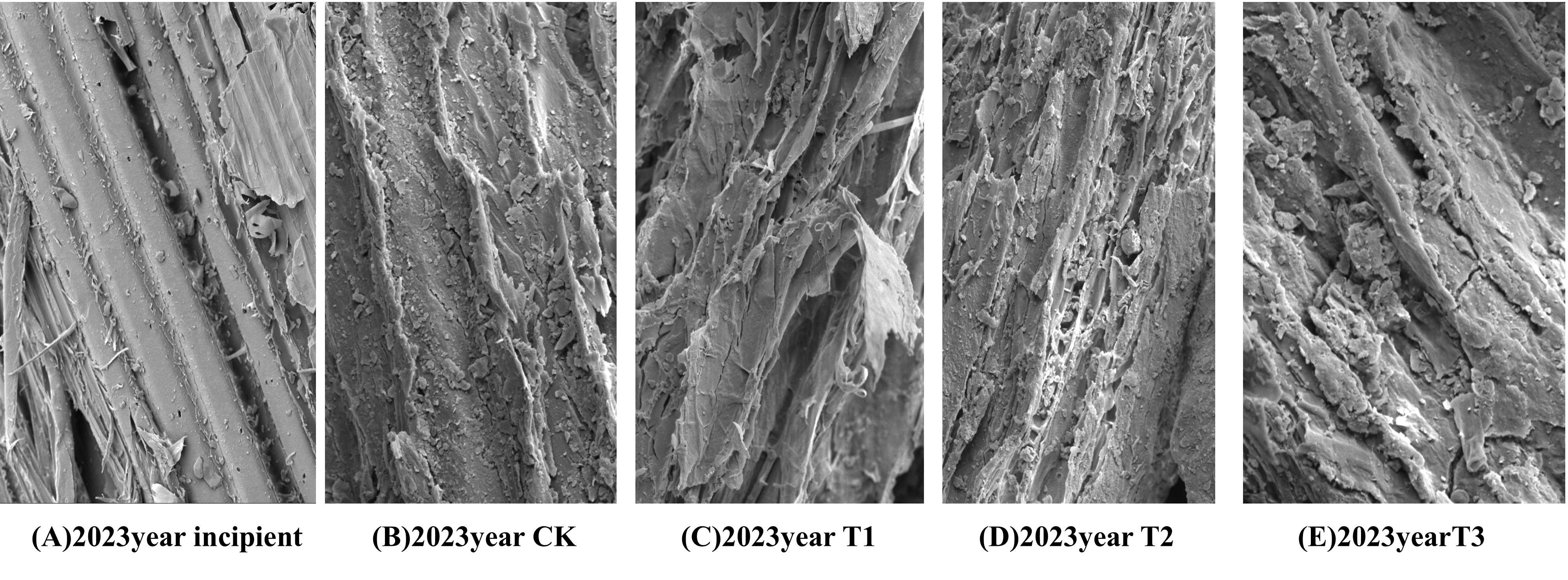 |
| --- |
| 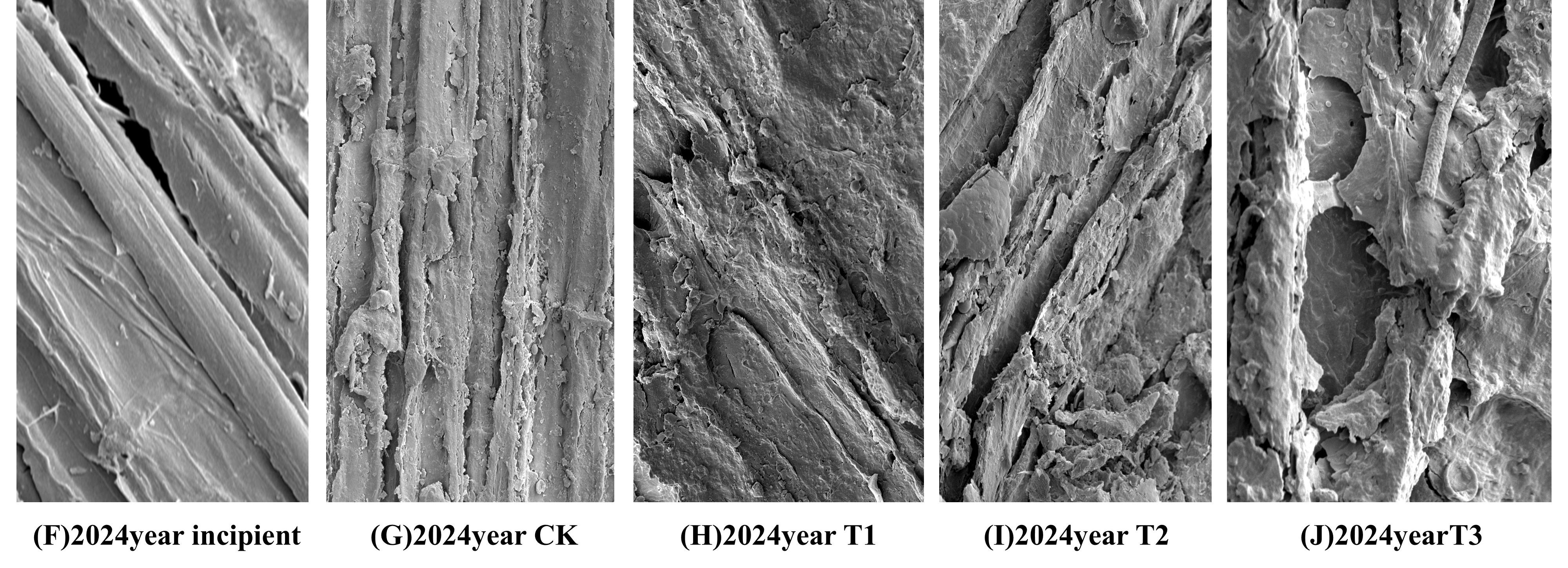 |
| **Figure 9.** Scanning electron microscopy of straw surface structure at maturity stage in two years. |

| 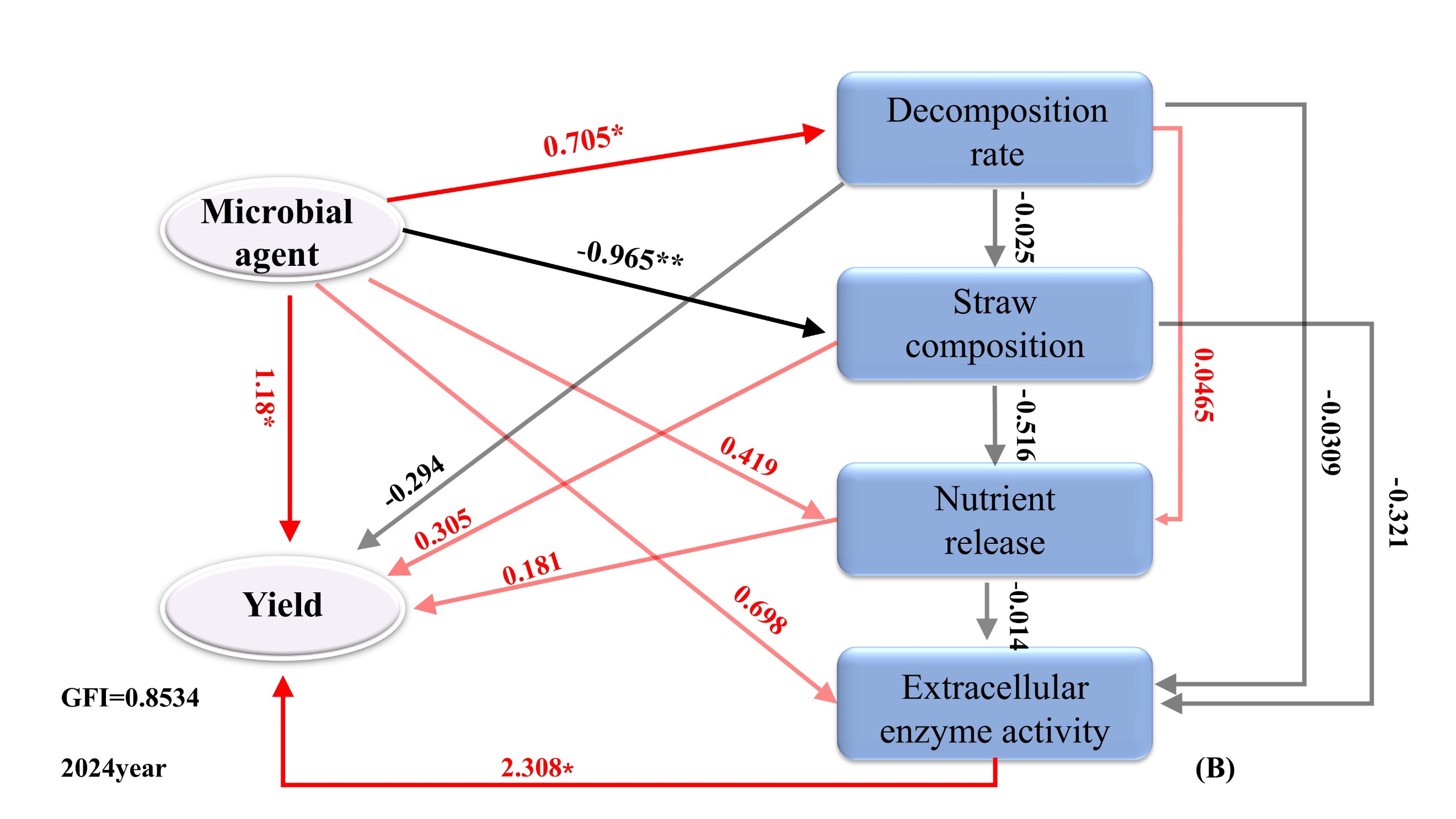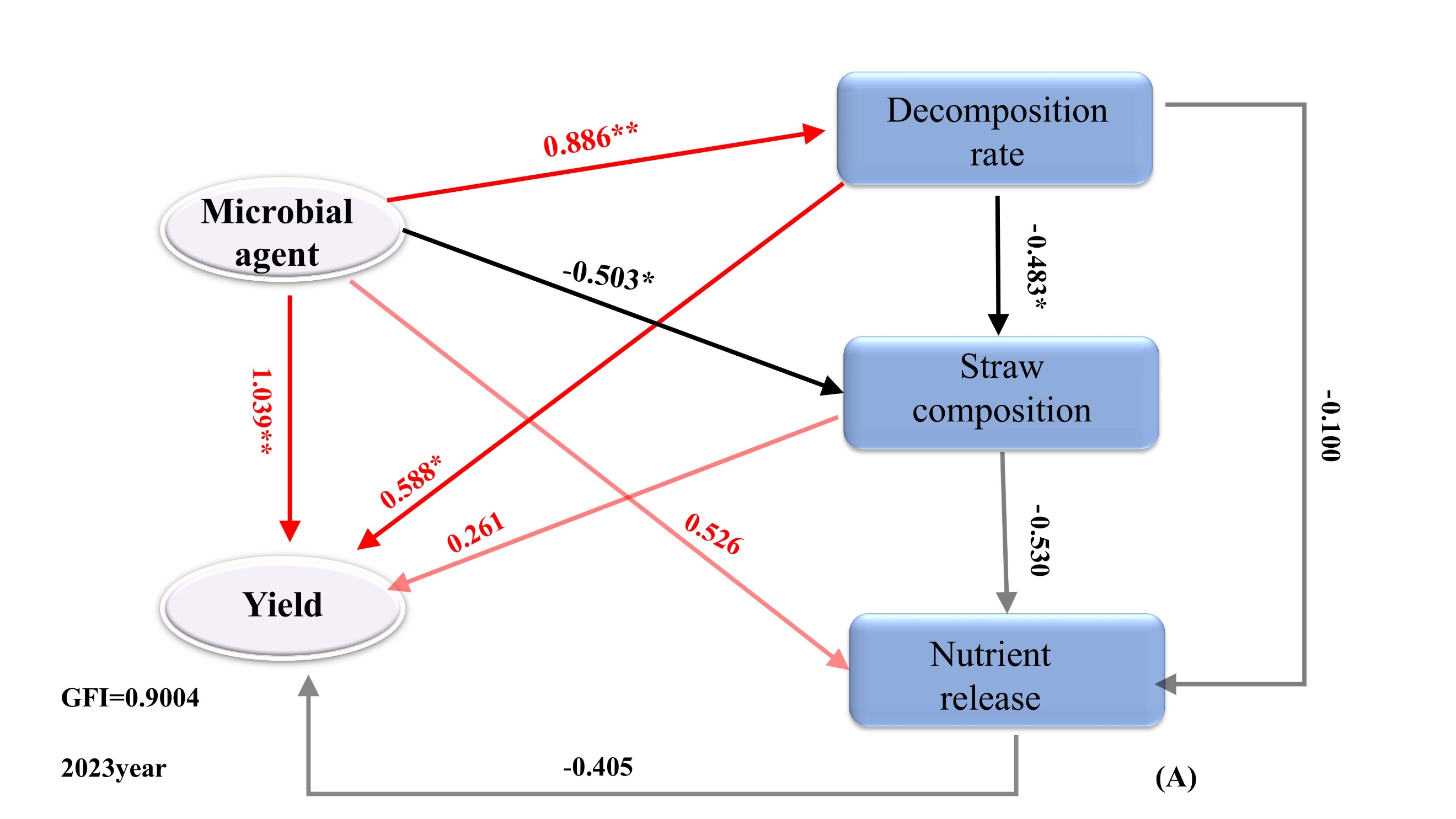 |
| --- |
| **Figure 10.** Partial least squares path model structural equation model (PLS-PM) that affects each index after adding different bacteria.  Note: Numbers next to the arrows indicate path coefficients, with red indicating a positive effect and black indicating a negative effect. * and ** indicate that the correlation reaches the 0.05 and 0.01 significance levels, respectively. |
| 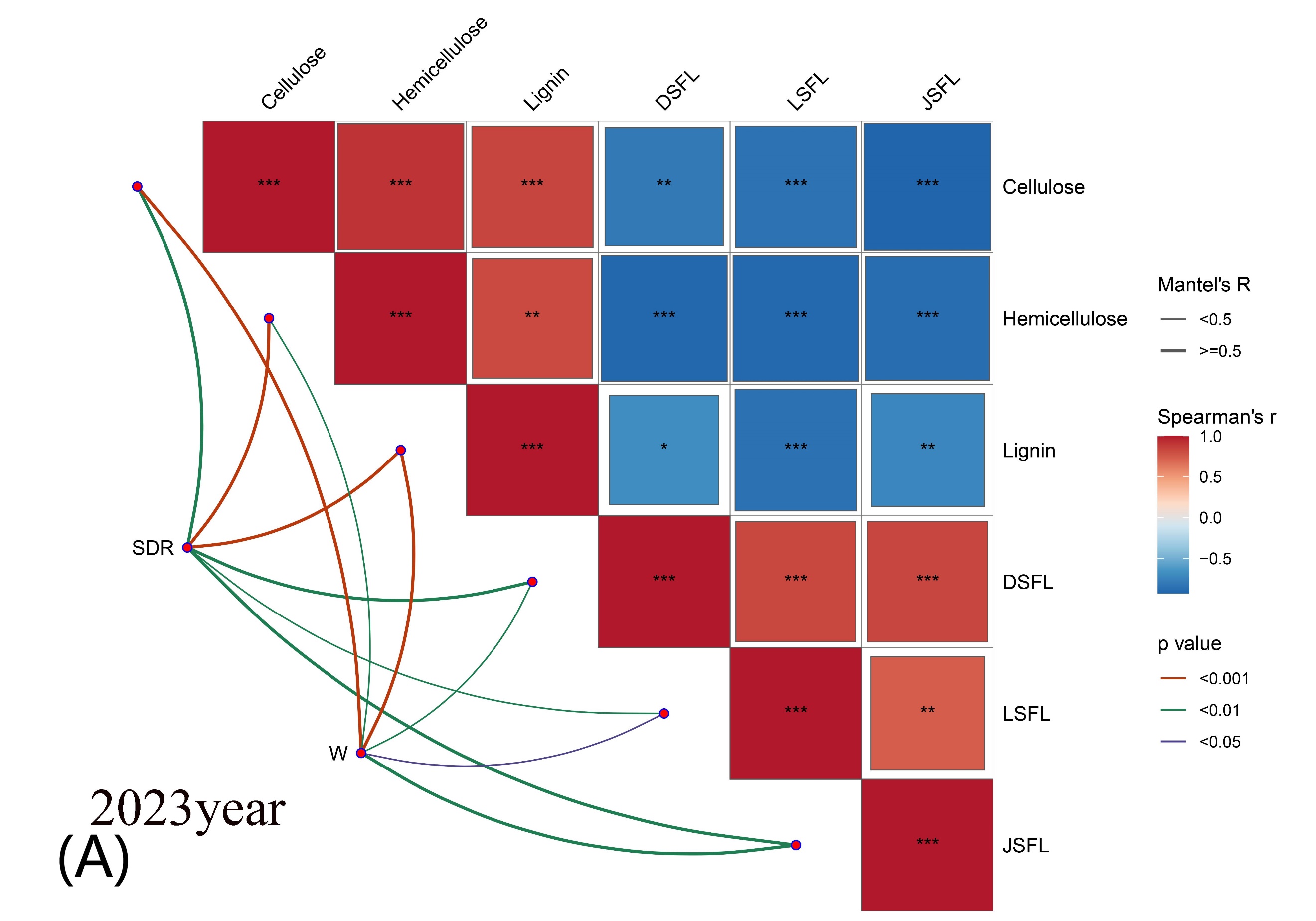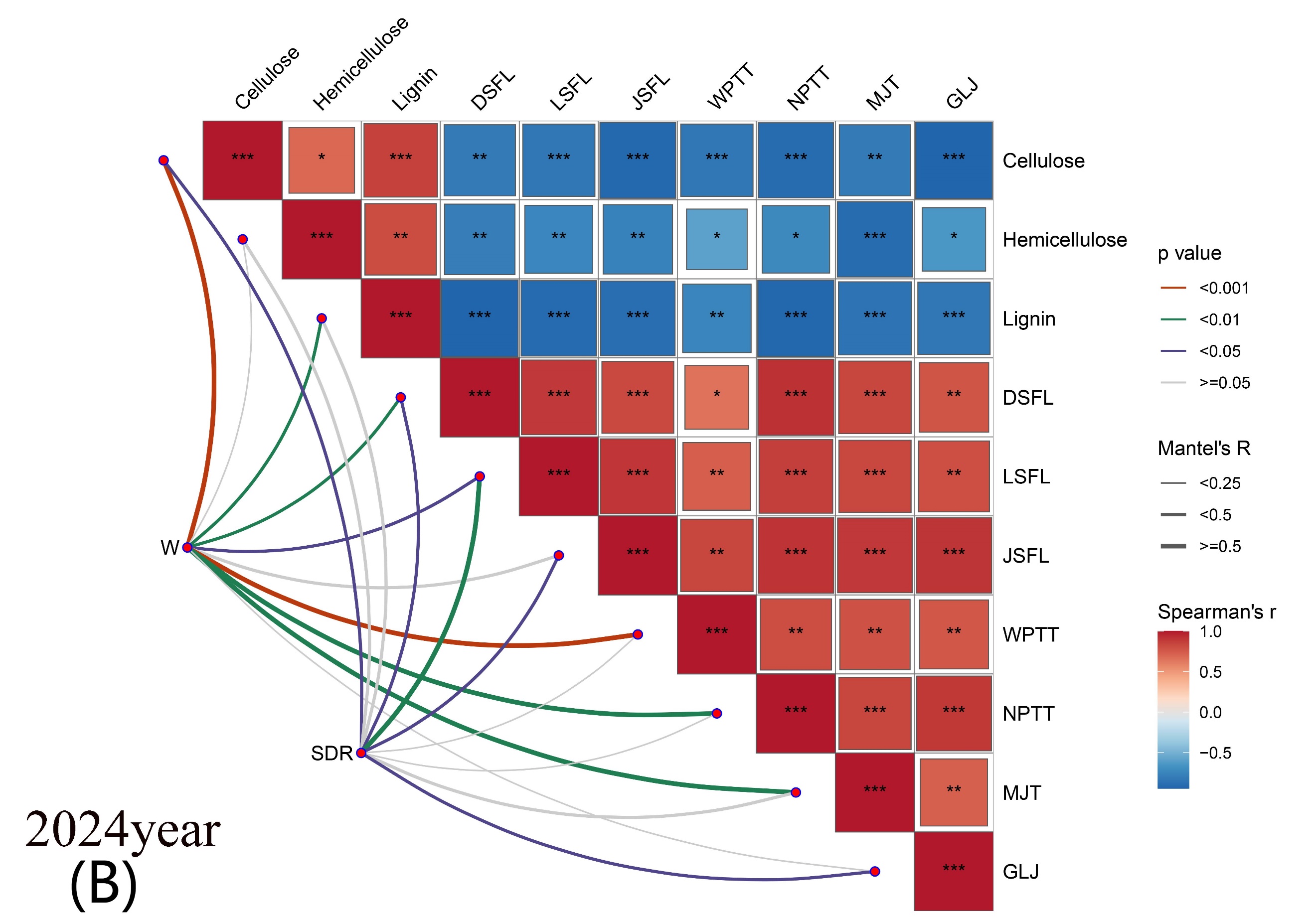 |
|  |
| **Figure 11.** Mantel-test correlation analysis heat map of each index after adding different microbial agents  Note: SDR: straw decomposition rate; W: single plant weight; DSFL: straw nitrogen release rate; LSFL: straw phosphorus release rate; JDFL: straw potassium release rate; WPTT: exo-β-1,4-glucanase; NPTT: endo-β-1,4-glucanase; MJT: xylanase; GLT: β-mannanase. |

## Supplementary Tables

**Table 1** physicochemical properties of the test soil

| Year | pH | OM  (g/kg) | AP  P_2_O_5_(mg/kg) | AK  K_2_O(mg/kg) | AN  N(mg/kg) | Conductivity (us/cm) |
| --- | --- | --- | --- | --- | --- | --- |
| 2023 | 8.73 | 25.47 | 28.40 | 136.67 | 92.06 | 420.17 |
| 2024 | 8.95 | 31.29 | 29.50 | 118.00 | 123.17 | 464.00 |

**Table 2** Initial nutrient content of test straw

| Year | TN | TP | TK | TC | C/N | Cellulose | Hemicellulose | Lignin |
| --- | --- | --- | --- | --- | --- | --- | --- | --- |
|  | (mg/g) | (mg/g) | (mg/g) | (mg/g) | (mg/g) | (mg/g) | (mg/g) | (mg/g) |
| 2023 | 8.03 | 2.25 | 15.45 | 614.8 | 77.0 | 281.5 | 352.0 | 158.7 |
| 2024 | 5.00 | 6.87 | 24.44 | 356.7 | 72.0 | 327.5 | 369.0 | 229.5 |

**Table 3** Effects of different preservatives on soybean yield and its components

| year | Treatment | Weight per plant | 100-grain weight | pod number per plant | theoretical yield |
| --- | --- | --- | --- | --- | --- |
| 2023 | CK | 43.44±2.01c | 18.81±0.42b | 23.11±0.51b | 8.24±0.12c |
|  | T1 | 49.67±3.00b | 19.07±0.57ab | 23.44±2.69b | 9.47±0.37b |
|  | T2 | 52.67±2.96a | 19.30±0.3ab | 24.56±1.84b | 10.17±0.2a |
|  | T3 | 54.11±3.01a | 19.44±0.38a | 29.11±1.17a | 10.54±0.32a |
| 2024 | CK | 38.22±2.83c | 18.05±0.26a | 17.06±0.52c | 6.90±0.47b |
|  | T1 | 39.56±3.15bc | 18.19±0.56a | 19.74±0.87b | 7.20±0.24b |
|  | T2 | 40.44±2.92b | 18.21±0.51a | 22.39±0.42a | 7.36±0.56b |
|  | T3 | 46.69±3.03a | 18.37±0.22a | 22.41±0.5a | 8.58±0.66a |

**Note: Different lowercase letters indicate significant differences between treatments(*P<0.05*).**
